# Supplementary material for: Spectroscopic identification of active sites for the oxygen evolution reaction on iron-cobalt oxides
Source: Nat Commun. 2017 Dec 8;8:2022. doi: 10.1038/s41467-017-01949-8 (PMC5722881; doi:10.1038/s41467-017-01949-8)
Supplement: Supplementary file 1 — Supplementary Information [file 41467_2017_1949_MOESM1_ESM.pdf]

Supplementary Note 1. Electrochemical Analysis.

Cyclic voltammetric (CV) characterization of the binary Fe-Co series revealed irreversible anodic features in the initial voltammetric cycle for all Co-containing samples examined here (CVs acquired during *in-situ* XAS experiments shown in Fig. 1 and Supplementary Figs. 1 and 15). The current density associated with the irreversible features was found to decrease, and the peaks to shift anodically, as the Fe-content of the film increased. The irreversible portion of the current density was isolated for each catalyst composition (Fig. 1b and Supplementary Fig. 1c) by subtraction of current density measured during the fifth voltammetric cycle from the initial cycle. This irreversible portion of the difference voltammograms was fitted with a series of Gaussian-shaped peaks for **100% Co**, **88% Co** and **75% Co** (Fig. 1b). Resolution of the individual redox features in the remaining films was impeded by decreased currents and convolution of the redox processes (Supplementary Fig. 1). The charge densities associated with the irreversible oxidation processes were calculated by integration of the difference voltammograms in the appropriate voltage range (Supplementary Table 1).

Steady state electron transfer kinetics were measured immediately following completion of voltammetric cycling. Measurements were performed by a series of chronoamperometric experiments wherein the voltage was increased from 1.0 V to 1.6 V *vs*. RHE in +10 mV steps with 60 second increments. Tafel plots were prepared by plotting the logarithm of current density at the end of the 60 second steps against overpotential (Supplementary Fig. 2). Tafel plots for **100% Co** contained a single linear region while all others exhibited two regions. The fit parameters for each linear region, along with the voltage and current density at which transitions occurred, are provided in Supplementary Table 2. A second CV was recorded immediately following the chronoamperometric experiments and compared to the initial CV (not shown). In all cases the second CV was found to be superimposable with the final cycle of the initial CV, confirming both catalyst stability and the achievement of steady state behavior in the initial CV.

Uncompensated resistance was numerically corrected for during data processing for all electrochemical experiments. Resistance compensation was not utilized during data acquisition.

Supplementary Note 2. X-ray absorption spectroscopy.

X-ray absorption spectroscopy (XAS) were carried out at the KMC-3 beamline at the BESSY II facility in Berlin, Germany. Data for X-ray absorption fine-structure spectroscopy (XAFS) and X-ray absorption near-edge structure (XANES) analyses were acquired by scanning the incident X-ray energy through the Fe and Co K-edge regions while the fluorescence spectra and calibration data were simultaneously recorded using a previously described experimental apparatus.[1,2] High purity metal foils served as calibration standards (10 μm thick Fe or Co foils, >99.99% purity, Goodfellow Cambridge

Limited). A liquid helium cooled cryostat kept the sample chamber temperature at 20 K during XAFS data acquisition. *In-situ* spectroelectrochemical experiments were performed at room temperature using a custom-built Teflon cell that has been previously described.[2] *In-situ* measurements were performed in fluorescence mode using back-side illumination.

Catalyst films were deposited on 100 μm thick GC wafers as described in the main text. As-prepared samples were stored in liquid N_2 prior to XAS analysis. *Quasi in-situ* samples were prepared using a freeze-quench method,[2] where samples mounted in a custom-made thin-layer electrochemical cell were submerged in liquid N_2 while under applied voltage bias and subsequently stored in liquid N_2 until measurements were performed.

Analysis of the XANES region of spectra was carried out on data acquired at 20 K. The Fe K-edge exhibited negligible changes throughout the dataset. The Co K-edge exhibited a negative shift as Fe-content was increased, and a positive shift when oxidizing voltages were applied (Supplementary Fig. 3). Attempts to quantify the differences in K-edge location were made by comparison to crystalline standards. The XANES for Co and Fe crystalline reference compounds show linear correlation between the oxidation state of the metal ion and the energy difference between the pre-edge feature and the K-edge location (Supplementary Figs. 3a and 3d). The average oxidation state of Fe and Co ions in the catalyst films are estimated using calibration curves generated from crystalline reference compounds. The energy difference between the pre-edge feature and the K-edge was measured for all samples and reference compounds by first shifting the spectra such that the pre-edge feature was located at 0 eV, then extracting the K-edge location. The leading-edge of the pre-edge feature was fitted with an arctan function and the mid-point of the fit line used to shift the spectra to 0 eV. The relative location of the K-edges were then measured using a previously described integral method ($0.15 < \mu < 1.0$).[3] Results indicate that Fe atoms experience no oxidation state change under the conditions utilized here, while the Co oxidation state is dependent on both Fe-content and voltage.

In-situ XAS-spectroelectrochemical experiments were performed on a selection of catalyst compositions (**100% Co** to **50% Co**) to track redox changes in response to electrochemical perturbations. The dependence of edge position on oxidation state induces a dependence of fluorescence intensity on oxidation state at any single incident photon energy within the steeply rising region of the K-edge (for example, see Supplementary Fig. 3c). To exploit this relationship, experiments were performed by maintaining a constant incident photon energy ($E_{\text{in-situ}}$) and recording changes in the intensity of the fluorescence as a response to electrochemical perturbations. An $E_{\text{in-situ}}$ of 7724 eV was chosen here as it resides in the steeply rising region of the Co K-edge for all samples. A series of spectroelectrochemical experiments were sequentially carried out on each electrode as depicted in Supplementary Fig. 6. The testing protocol included (i) testing material stability by periodic acquisition of XANES spectra while

catalyst films were held at 0.89 V *vs* RHE (*in-situ* XANES), (ii) acquisition of a series of XANES spectra while the catalyst films were held at selected voltages to enable calibration of the data (*in-situ* XANES), (iii) a cyclic voltammogram (*in-situ* XAS-CV) and (iv), chronoamperometric measurements where the voltage was stepped between 1.30 and 1.60 V *vs* RHE (*in-situ* XAS-CA).

In-situ XANES edge spectra indicated a high degree of film stability and a linear correlation between the fluorescence intensity and location of the Co K-edge in Co-rich samples. The raw XANES spectra for **100% Co** (Supplementary Figs. 7a and 7b; I_0 is the intensity of incident X-rays) reveal a reproducible baseline in the pre-edge region between 7600-7700 eV, confirming a stable background signal, and the expected decrease in fluorescence intensity upon oxidation of the film at 1.60 V *vs*. RHE. The four spectra acquired at 0.89 V *vs*. RHE are superimposable, indicating negligible loss of cobalt ions from the film over the entire course of experiments. Traditional processing of the XANES data, which involves background subtraction and normalization of the post K-edge region to unity, yields spectra that exhibit excellent agreement with the raw data (Supplementary Fig. 7c and 7d). Similar behavior was observed for **88% Co** and **75% Co** (not shown). Further addition of Fe to the catalyst films resulted in a decrease in baseline fluorescence following application of oxidizing voltages. The raw XANES spectra for **50% Co** (Supplementary Figs. 8a and 8b) show a baseline (7600-7700 eV) that drifts to lower values with time. The magnitude of the baseline shift increases with the length of time that the catalyst film was held at 1.6 V *vs*. RHE. A simple baseline shift of the spectra yields spectra that are in excellent agreement with processed spectra (Supplementary Figs. 8c and 8d). These results, and the proximity of the Fe K-edge (*ca*. 7112 eV), lead us to attribute the baseline drift to the dissolution of Fe from the film.

Calibration curves were generated from *in-situ* XANES spectra to enable conversion of the fluorescence intensity into changes in the location of the Co K-edge. Plots of the normalized intensity of fluorescence at 7724 eV against the edge position confirm a linear correlation exists for all samples examined (Supplementary Fig. 9). Plots of the raw fluorescence against the edge position (extracted from processed spectra) yield similar linear correlations. The observed drift in fluorescence baseline for **62% Co** and **50% Co** results in deviation from linearity for the data points acquired following acquisition of the XANES spectra at 1.6 V vs. RHE. These points were therefore not utilized in the calibration curves. The linear slopes of Raw Fluo/I_0 *vs*. Edge Position plots (Supplementary Fig. 9) were employed to convert changes in fluorescence intensity into changes in edge position (ΔE_{edge}) for the *in-situ* XAS-CV and *in-situ* XAS-chronoamperometry experiments for each sample. The experimental setup utilized for *in-situ* experiments precluded simultaneous measurement of the sample and a calibration standard as done in XANES measurements at 20 K. We therefore limit the analysis of the *in-situ* data to changes in the Co K-edge location (ΔE_{edge}), without assigning specific oxidation states.

Comparison of the spectral component of *in-situ* XAS data with the electrochemical component enables interpretation of electrochemical results in terms of Faradaic and catalytic components. Shifts in ΔE_{edge} were observed to coincide with current flow during *in-situ* XAS-chronoamperometry (Supplementary Figs. 10-14) and *in-situ* XAS-CV (Supplementary Fig. 15) experiments for all films examined. The first derivative of ΔE_{edge} with respect to time ($d\Delta E_{edge}/dt$) yields the rate at which the Co K-edge shifts with time, in units of eV s^{-1}. The linear relationship between E_{edge} and the average oxidation state of Co atoms (Supplementary Fig. 3) indicates that these plots are proportional to the rate of change in oxidation state with time. Qualitative comparisons of such derivative plots with electrochemical current flow can thus offer insight into the portion of electrochemical current due to Faradaic processes. The fluorescence signal contained random fluctuations in ΔE_{edge} over a multi-second timeframe. Contributions from this noise were minimized for *in-situ* XAS-CA data by performing multiple potential step cycles and averaging the results. The desire to analyze the irreversible redox processes during *in-situ* XAS-CV experiments precludes such averaging. Data shown was therefore mathematically smoothed using a weighted moving-average to approximate a 1 second acquisition time.

An exponential dependence of ΔE_{edge} on time was observed for both the anodic and cathodic voltage-steps on **100% Co** (Supplementary Figs. 10a and 10b), with the decrease in ΔE_{edge} during cathodic steps indicating a net reduction of Co ions and an increase during anodic steps indicating a net oxidation of Co ions. Comparison of *i vs. t* plots with $d\Delta E_{edge}/dt$ *vs. t* reveals excellent agreement between the two signals for cathodic voltage steps (Supplementary Fig. 10c), suggesting that the current flow is purely Faradaic in nature. Anodic voltage steps reveal separation of the current and spectral signals after *ca*. 0.5 seconds, which can be readily interpreted as a transition from Faradaic currents at early times into steady state catalytic currents at longer times. *In-situ* XAS-CV on **100% Co** revealed a minor shift in ΔE_{edge} between *ca*. 1.0 and 1.1 V *vs*. RHE and a major shift between *ca*. 1.4 and 1.6 V (Supplementary Fig. 15a). The first derivative of the spectral signal ($d\Delta E_{edge}/dE$; Fig. 4) was able to resolve the major redox features in the electrochemical data, with a peak located at *ca*. 1.5 V on the anodic sweep and a peak at *ca*. 1.45 V with a shoulder at *ca*. 1.35 V on cathodic sweeps, but unable to resolve the comparably smaller irreversible redox processes.

In-situ XAS-chronoamperometric experiments revealed changes in both the spectral and electrochemical behavior upon introduction of Fe to catalyst films (Supplementary Figs. 11-14). A bi-exponential dependence between ΔE_{edge} and time emerged for all Fe-containing compositions. Electrochemical currents were once again traced by $d\Delta E_{edge}/dt$ during cathodic voltage steps, confirming that cathodic current flow is primarily Co-based and Faradaic in nature. Electrochemical data recorded during anodic voltage steps, however, consisted of an exponential decay in current density that transitioned to a slower exponential growth. Inspection of $d\Delta E_{edge}/dt$ reveals that a significant portion of

cobalt ions continue to be oxidized during the slow *exponential increase* in current density. The electrochemical data therefore represents of a convolution of at least two distinct redox processes, as discussed in the manuscript. Irreversible shifts in ΔE_{edge} were observed during the initial anodic sweep of *in-situ* XAS-CV experiments for all Fe-containing compositions, confirming the Co-based nature of these processes (Supplementary Fig. 15).

Supplementary Note 3. EXAFS Simulations.

X-ray absorption spectra were processed using an in-house software package. Following dead-time correction the fluorescence signals were divided by the intensity of the incident X-ray beam (I_0) and "deglitched" by removal of anomalous data points. Spectra were then calibrated by shifting the energy-axis such that the first peak in the first derivative of the X-ray absorption spectrum for the calibration standards was located at appropriately (7112 eV for Fe spectra, 7709 eV for Co spectra). Spectra were then shifted by subtracting a constant value such that the fluorescence in the pre-edge region was zeroed and the region beyond the K-edge jump was normalized to unity by dividing by a third-order polynomial fit line. Multiple spectra acquired per sample were then averaged. Oscillations in the spectrum were extracted by first minimizing a "knot spline" fit line to the spectra (5 knots between 7720 and 8270 eV for Co, 3 knots between 7123 and 7700 eV for Fe) and subtracting the fit from the data. The energy axis was shifted by subtraction of 7710 eV (Co spectra) or 7113 eV (Fe spectra). The data was then converted into *k*-space and the data was reduced to 160 equally spaced points by interpolation (*ca.* 0.075 A^{-1} spacing).

The oscillations in the extracted data can be described by contributions of a total of n_{shell} shells according to the equation:

$$\chi(k) = S_0^2 \sum_i^{n_{shell}} A(R_i, k)_i \cdot N_i \cdot e^{-2\sigma_i^2 \cdot k^2} \sin(2k \cdot R_i + \phi_i) \tag{S1}$$

Where individual coordination shells are defined to consist of an average of N_i atoms of a given element that are present at distance R_i from the absorbing atom. Variations in R_i of each shell are described by the Debye-Waller parameter (σ_i) and the sum of oscillations are dampened by an amplitude reduction factor (S_0^2). The scattering amplitude ($A(R_i,k)_i$) and phase correction (ϕ_i) for each shell were obtained by *ab-initio* calculations using Feff 8.4. Calculations were performed on absorbing atoms in each unique coordination environment for fragments of $LiCoO_2$, Co_3O_4 and Fe_2CoO_4 for cobalt analysis, and α-Fe_2O_3, α-FeO(OH), and Fe_2CoO_4 for iron analysis. Simulations of the extracted EXAFS oscillations were performed using SimXLite software, where the oscillations were k^3-weighted and simulated by least-squares fitting of the data in *k*-space. An S_0^2 value of 0.78 was found to yield an N_i of 6.0 for the Co-O shell in the spectrum exhibiting the most intense oscillations (*quasi in-situ* spectrum of **100% Co**) and was

applied to Co simulations for the entire composition series. Errors are estimated as previously described.[1] Spectra were fitted in the 3.0 to 12.0 Å^{-1} region between **100% Co** to **50% Co**, and from 3.0 to 11.0 Å for **38% Co** to **12% Co**. Due to the strong correlations between σ_i and N_i, the decision was made to fix σ_i values across the composition series. Debye-Waller (σ_i) values for Co-O and Co-Co shells were chosen to correspond with reported values for amorphous cobalt oxides in previous studies.[1,4-6]

Fourier-transform of the Co data revealed prominent features in three general regions for the composition series (Supplementary Fig. 4, Supplementary Table 3). The major feature at a reduced distance of *ca.* 1.5 Å represents Co-O vectors and that at *ca.* 2.5 Å provides the fingerprint of di-μ-oxo bridged Co-Co motifs.[4,5] These 2-coordination shells have repeatedly yielded successful fits for disordered cobalt oxides prepared by anodic electrodeposition (labeled **CoCat**).[1-2,4-6] The feature observed between *ca.* 3.0 and 3.5 Å in the data here has not been previously reported in amorphous cobalt oxides, hinting at structural differences. These differences are confirmed by comparisons between the XAFS oscillations for **100% Co**, $LiCoO_2$ and **CoCat** (Supplementary Fig. 16)**.** This comparison reveals differences in both oscillation intensity, indicative of a more disordered structure around cobalt ions in **100% Co**, and in interference patterns in three distinct regions. Attempts to simulate the data with a single Co-O coordination shell results in an $N_{Co\text{-}O} < 4.0$ Å, which is physically unrealistic. Releasing restraints on $\sigma_{Co\text{-}O}$ does little to help: $\sigma_{Co\text{-}O}$ values begin to move >0.07 Å and $N_{Co\text{-}O}$ values remain <5.0 (Supplementary Tables 5 and 6). Such models also produce poor numeric fits for the data; visual inspection of the XAFS data identifies an extremely poor fit for region 1 in Supplementary Figs. 17 and 18, where Co-O oscillations dominate the observed behavior. Simulations performed using two Co-O shells are therefore favored here. The di-μ-oxo bridged Co-Co motif is successfully described by a single Co-Co shell for **100% Co**. Addition of a second Co-M shell in this region was found to result in significant improvements to fit quality for Fe-containing compositions (see Supplementary Tables 5 and 6), with negligible differences between simulations with M as Fe or Co. Simulations resulted in reproducible R_i values, 2.84 ± 0.01 Å and 3.04 ± 0.4 Å, for the two coordination Co-M shells between **38% Co** and **88% Co**. The consistency in bond distances across the composition series, combined with the excellent match in interatomic distance with literature values, boosts confidence in the validity of a two-shell simulation of this region for Fe-containing samples. A single Co-Co shell provides a match for the third feature across the composition series. Visual inspection of the data in Supplementary Figs. 17 and 18 reveal that inclusion of this final shell enables the model to capture the unique features in the data that prevent the use of a layered-double hydroxide model: a small oscillation in region 2 and a plateau in region 3.

The oscillations observed in Fe EXAFS spectra exhibit significant dampening relative to the Co K-edge region, as has been previously reported for amorphous iron-oxide.[7] Simulations of the Fe spectra were therefore limited to the *k*-3 to *k*-10 region across the series with a fixed S_0^2 value of 0.78. Fixed σ_i

values were utilized (0.076 Å for Fe-O shells, 0.078 Å for Fe-M shells), chosen to be in agreement with previously reported values.[7,8] Simulation of the **0% Co** sample (pure FeO_x) was initiated using a single Fe-O and Fe-Fe shell, but to obtain physically realistic structural parameters addition of a second Fe-O shell was required. Addition of a Fe-Fe shell at 3.50 Å yielded significant improvements in fit quality (Supplementary Fig. 5 and Supplementary Table 4). The shells comprising the structural model for **0% Co** thus include Fe^{III}-O (R = 1.96 Å, N = 4.9), Fe^{III}-OH (R = 2.13 Å, N = 0.8), di-μ-oxo bridged Fe^{III}-Fe^{III} octahedra (R = 3.02 Å, N = 1.9) and a bridging, corner-sharing Fe^{III} octahedra (R = 3.50 Å, N = 0.9). Assignments are supported by previous reports of a mixture of edge and corner-sharing Fe-octahedra in photochemically deposited iron oxide,[7,9] by interatomic distances that match those present in XRD patterns for hematite,[7] and by the XANES analysis above indicating exclusively Fe^{III} in the films. Compositions containing cobalt were simulated with an additional Fe-Co shell (Fig. 3 and Supplementary Fig. 5, Supplementary Table 4) to enable comparison with the Co K-edge data. The coordination number for the fifth shell increases with Co-content, mirroring the behavior observed for di-μ-oxo bridged Co^{II}-Fe^{III} motifs in the Co K-edge spectra, but the simulated bond length is not in agreement with the Co K-edge results. We note that the narrower range of XAFS data available for the Fe K-edge data decreases confidence in this model, and the number of free parameters introduces the risk that this model over-interprets the data. The Fe data was therefore not utilized for quantitative trend analysis. Stark differences between the FT-XAFS results for Co and Fe edges (Fig. 3a-3b) rule out geometric strain effects recently observed in an Fe-Ni LDH series.[8]

Supplementary Note 4. Visible-light Spectroelectrochemistry.

Spectroelectrochemical measurements were performed using a three-electrode setup and an in-house apparatus. A lock-in amplifier (Ithaco NF 3961B) connected to the Biologic SP300 potentiostat enabled simultaneous recording of spectral and electrochemical data. The amplifier synchronized a waveform generator (Wavetek, Model 180), which applied a 5 kHz AC-voltage to an LED bulb, with a photodiode detector to measure the intensity of light that passed through the sample. A pinhole shutter and lens focused the incident beam on the catalyst surface. A platinum wire counter electrode, a Hydroflex® RHE reference electrode and a catalyst-coated piece of FTO-coated glass were submerged in a quartz cuvette (20 x 20 x 50 mm) containing *ca*. 8 mL of electrolyte solution. Spectral results are presented as changes in absorbance (ΔA) at the given wavelength, defined as:

$$\Delta A(t) = \log_{10}\left(\frac{T_0}{T(t)}\right) \quad \text{(S2)}$$

Where the initial transmittance (T_0) was measured on the assembled apparatus, with the catalyst-coated FTO working electrode in the beam path, immediately before initiation of electrochemical experiments.

Supplementary Note 5. Extraction of Parameters for Analysis of Trends

Four irreversible peaks are resolved in difference CVs for **88% Co,** with apparent anodic shifts in $E_{p,1}$, $E_{p,3}$ and $E_{p,4}$ relative to **100% Co** (Fig. 1b). Further addition of Fe results in the disappearance of two peaks, leaving only two resolved irreversible processes in **75% Co**. The major irreversible feature, $E_{p,2}$, appears insensitive to Fe-content and remains constant at *ca*. 1.18 V. Assuming that the lowest voltage process in **100% Co** and **88% Co** are due to the same process, $E_{p,1}$ shifts anodically from 0.97 V to 1.08 V. Extrapolation of the *ca*. 10 mV per %-Fe shift predicts a peak at 1.22 V in **75% Co** and 1.35 V in **62% Co**. The shoulder located at 1.23 V in **75% Co** and the broad peak at 1.36 V in **62% Co** (Supplementary Fig. 1) are therefore attributable to the same process responsible for $E_{p,1}$. The high voltage peak, $E_{p,3}$, shifts from 1.41 V in **100% Co** to 1.43 in **88% Co** before merging into the catalytic OER wave in **75% Co**. The *ca*. 2 mV per %-Fe anodic shift suggested by the two data points tracks that observed for the reversible redox process, which is resolvable by spectroelectrochemical measurements until **62 % Co** (see below). Assignment of the irreversible shoulder at 1.25 V in **88% Co** to the same process as the shoulder in **100% Co** suggests an anodic shift of >20 mV per %-Fe for $E_{p,4}$. Failure to observe this process in other samples is explained by a shift beyond the onset of OER. Although convolution of the redox peaks inhibits accurate calculation of charge density passed per redox feature, integration of difference CVs provides reasonable estimates of charge density attributable to $E_{p,1}$ and $E_{p,2}$ (Supplementary Table 1).

Data acquired during *in-situ* XAS-CV experiments was used for analysis of trends between reversible and irreversible redox processes and specific structural motifs (Fig. 5a). Irreversible charge density was calculated for each composition by integration of the regions of interest in difference voltammograms (Supplementary Table 1 and Supplementary Fig. 15). Shifts in ΔE_{edge} of the Co K-edge are expected to contain contributions from baseline drift due to Fe-dissolution ($\Delta E_{edge,\ Fe\ loss}$), reversible redox processes ($\Delta E_{edge,rev.}$), and irreversible redox processes ($\Delta E_{edge,irrev.}$). To minimize errors due to noise in the fluorescence signal, contributions from these three processes were deconvoluted by comparison of changes to ΔE_{edge} over sequential cycles of the CV experiments, as depicted in Supplementary Fig. 15. The contribution from each component were defined as: (i) $\Delta E_{edge,Fe\ loss}$ was taken as the difference between ΔE_{edge} measured at the cathodic limits of sequential cycles, (ii) $\Delta E_{edge,rev.}$ was taken as the difference between the maximum ΔE_{edge} during a cycle and the value at the cathodic limit of the cycle, and (iii) $\Delta E_{edge,irrev.}$ was estimated by subtraction of $\Delta E_{edge,Fe\ loss}$ and $\Delta E_{edge,rev.}$ from the maximum ΔE_{edge}.

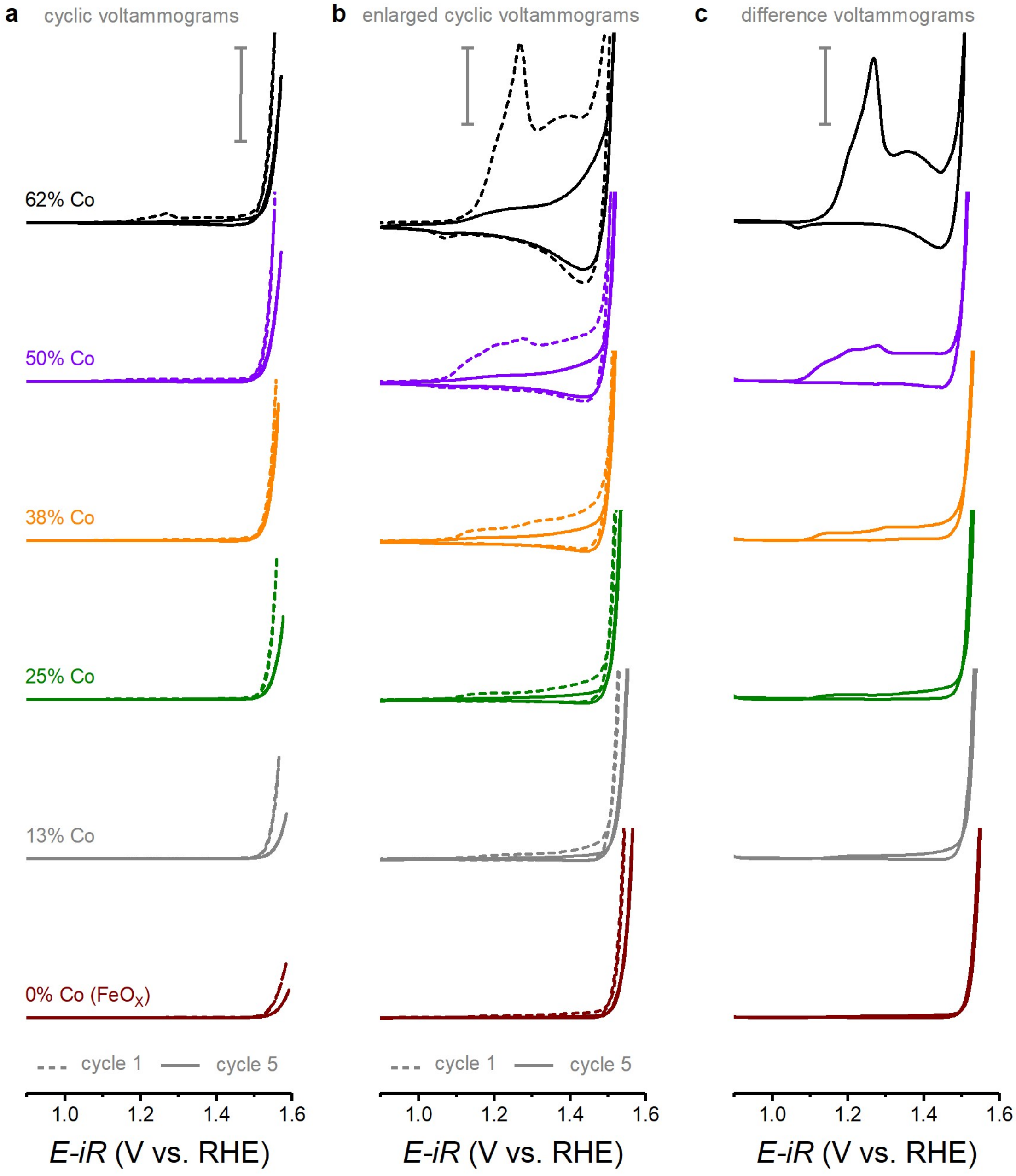

Supplementary Figure 1. Voltammetric behavior of Fe-Co oxide series. a and b, Two magnifications showing the first and fifth voltammetric cycles recorded at 10 mV s^{-1} in 1 M KOH. **c,** Difference voltammograms obtained by subtraction of cycle 5 from cycle 1. Data shown here for **62% Co** to **0% Co, 100% Co** to **75% Co** are included in the main text. Scale bars represent 5 mA cm^{-2} (**a**) and 0.2 mA cm^{-2} (**b** and **c**).

Supplementary Table 1. Charge density values calculated for irreversible oxidation processes.

Composition % Co	*E* (lower limit) V vs RHE	*E* (upper limit) V vs RHE	Q_{irrev} [a] mC cm^{-2}	Q_{irrev} / χ_{Co} [b] mC cm^{-2}
100	0.926	1.333	0.80	0.80
88	0.975	1.346	2.10	2.39
75	1.009	1.352	2.48	3.31
62	1.130	1.448	3.80	6.13
50	1.040	1.470	1.16	2.32

[a] irreversible charge calculated by integration of difference voltammograms between the given *E* limits (see also Supplementary Fig. 13)

[b] values normalized to 100% Co (χ_{Co} = %Co / 100)

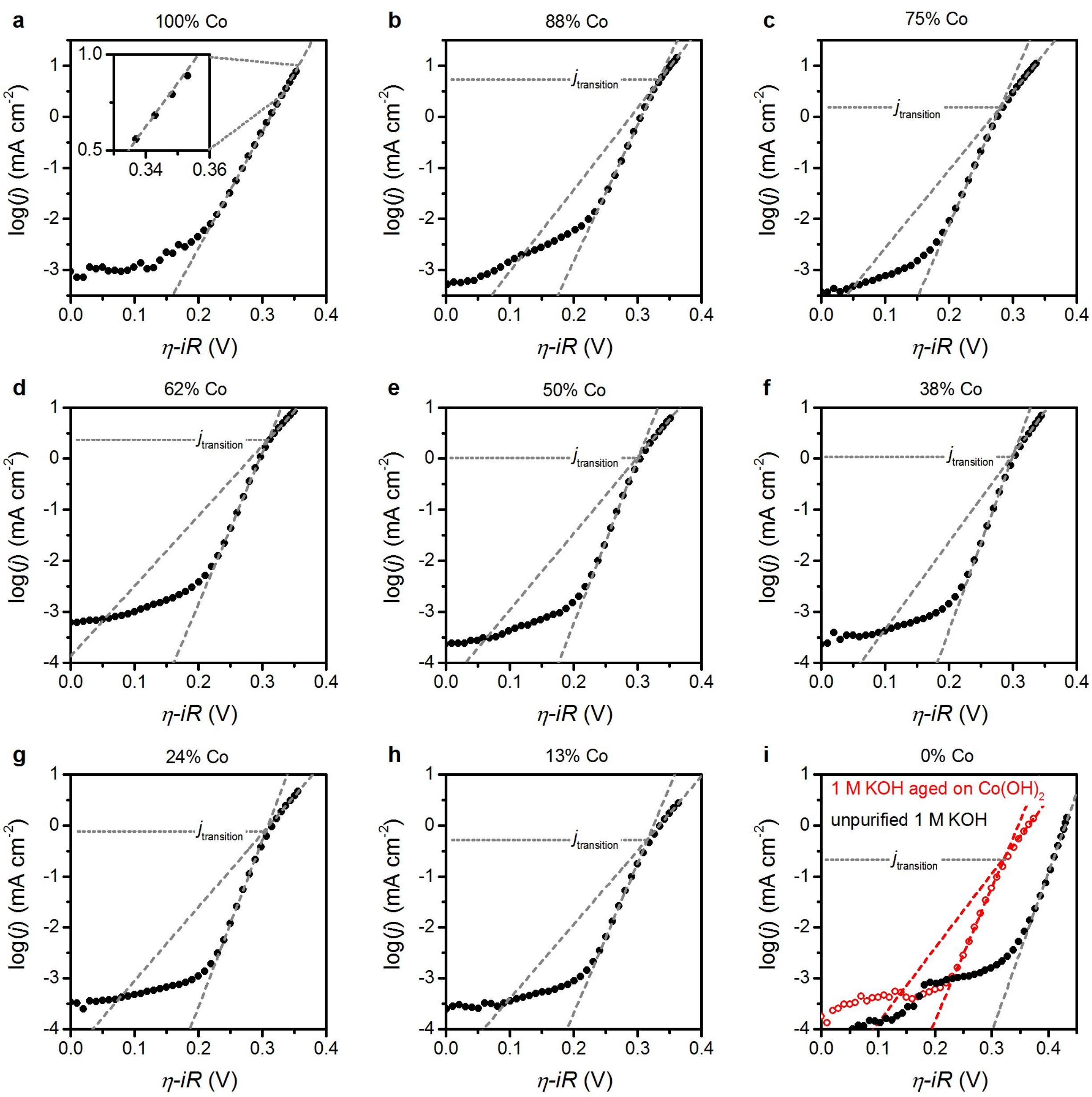

Supplementary Figure 2. Steady state electron transfer kinetics of the Fe-Co oxide composition series. Tafel plots for the oxygen evolution reaction by **a,** 100% Co, **b,** 88% Co, **c,** 75% Co, **d,** 62% Co, **e,** 50% Co, **f,** 38% Co, **g,** 24% Co, **h,** 13% Co and **i,** 0% Co in two different electrolyte solutions. The two linear components are marked by dashed trend lines, and their intersection is labelled $j_{\mathrm{transition}}$. Current density and voltage at the transition point are tabulated in Supplementary Table 2.

Supplementary Table 2. Electrocatalytic OER performance parameters for the Fe-Co oxide composition series

Composition % Co	Tafel slope[a] mV dec^{-1}	$\log(j_0)$ [a] mA cm^{-2}	Tafel slope[b] mV dec^{-1}	$\log(j_0)$ [b] mA cm^{-2}	$j_{transition}$ mA cm^{-2}	$E_{transition}$ V vs. RHE
100	44.2	-7.07	-	-	7.74[c]	
88	37.4	-8.16	62.4	-4.63	4.64	1.561
75	34.9	-7.84	65.4	-4.09	1.57	1.511
62	33.8	-8.77	72.9	-3.86	2.34	1.538
50	31.0	-9.67	67.0	-4.45	1.14	1.532
38	29.3	-10.19	58.5	-5.04	1.26	1.531
24	30.5	-10.10	69.0	-4.49	0.92	1.537
13	33.8	-9.60	68.7	-4.85	0.57	1.546
0[d]	31.9	-13.44	-	-	-	-
0[e]	38.2	-9.08	68.3	-5.34	0.26	1.554

[a] extracted from first linear region in Tafel plots (Supplementary Fig. 2)

[b] extracted from second linear region in Tafel plots (Supplementary Fig. 2)

[c] final data point in Supplementary Fig. 2a used as an estimate

[d] acquired using unpurified 1 M KOH_{aq}

[e] acquired using 1 M KOH_{aq} that was aged over $Co(OH)_2$

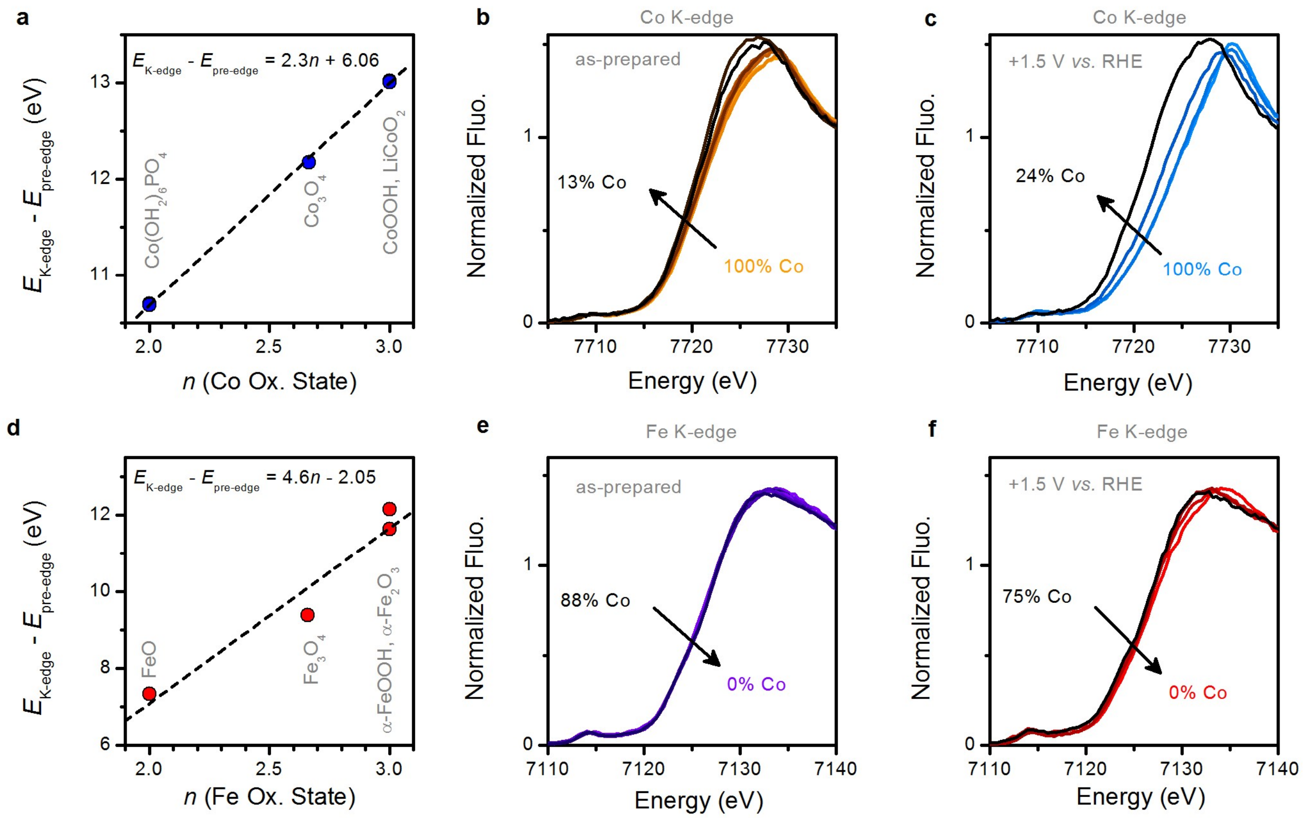

Supplementary Figure 3. X-ray absorption near-edge spectra in the Co and Fe K-edge regions. a, Calibration curve correlating the energy difference between the pre-edge and the Co K-edge location to oxidation state. **b, c,** Cobalt K-edge region for as-prepared and *quasi in-situ* samples. **d**, Calibration curve correlating the energy difference between the pre-edge and the Fe K-edge location into oxidation state. **e, f,** Fe K-edge region for as-prepared and *quasi in-situ* samples. Data shown for **100% Co**, **75% Co**, **50% Co** and **24% Co**.

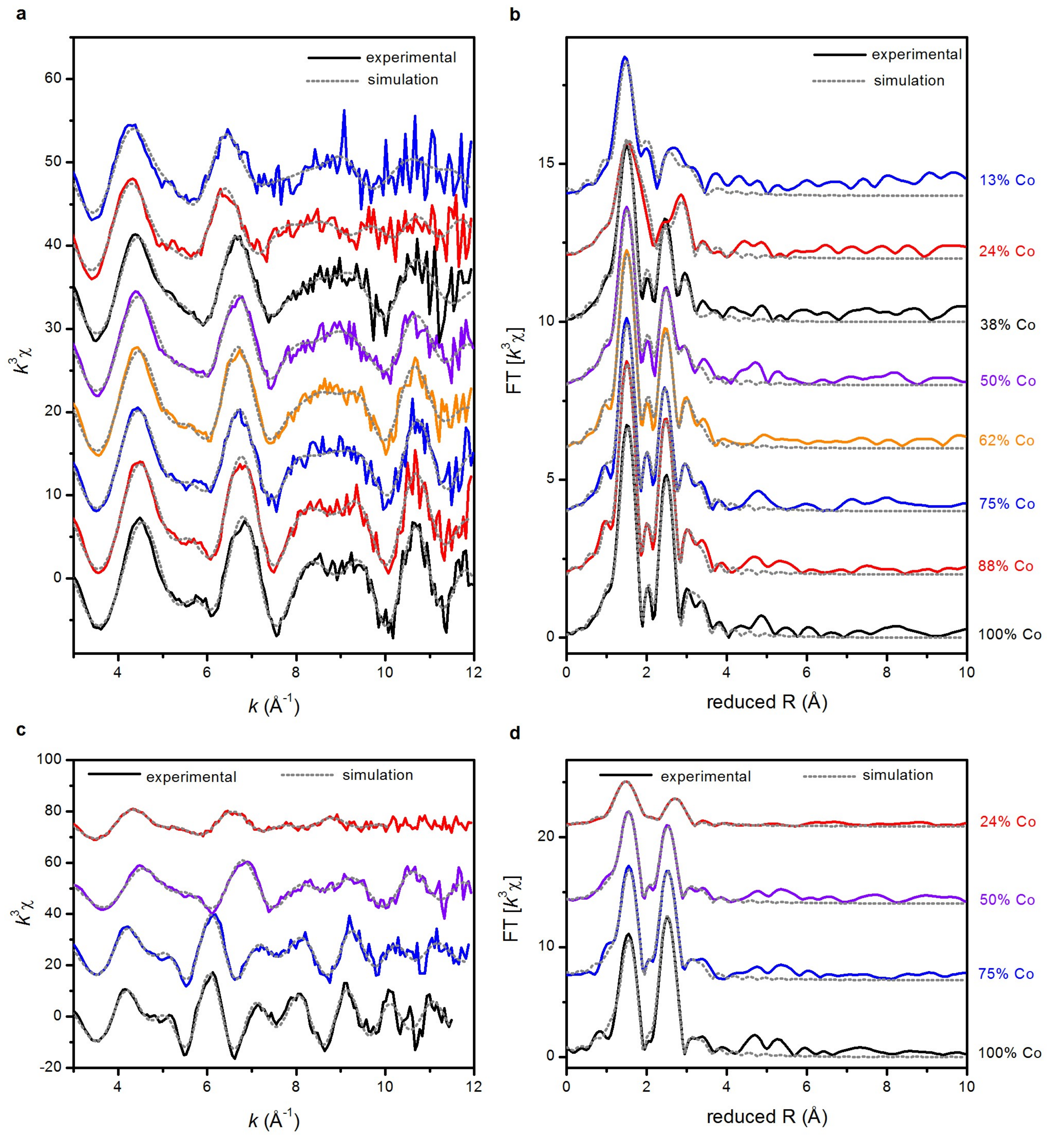

Supplementary Figure 4. X-ray absorption fine-structure spectra on the Co K-edge. Experimental data and simulation results in *k*-space and their Fourier transforms **a,b,** for as-prepared films and **c,d,** for *quasi in-situ* films frozen at 1.5 V vs. RHE.

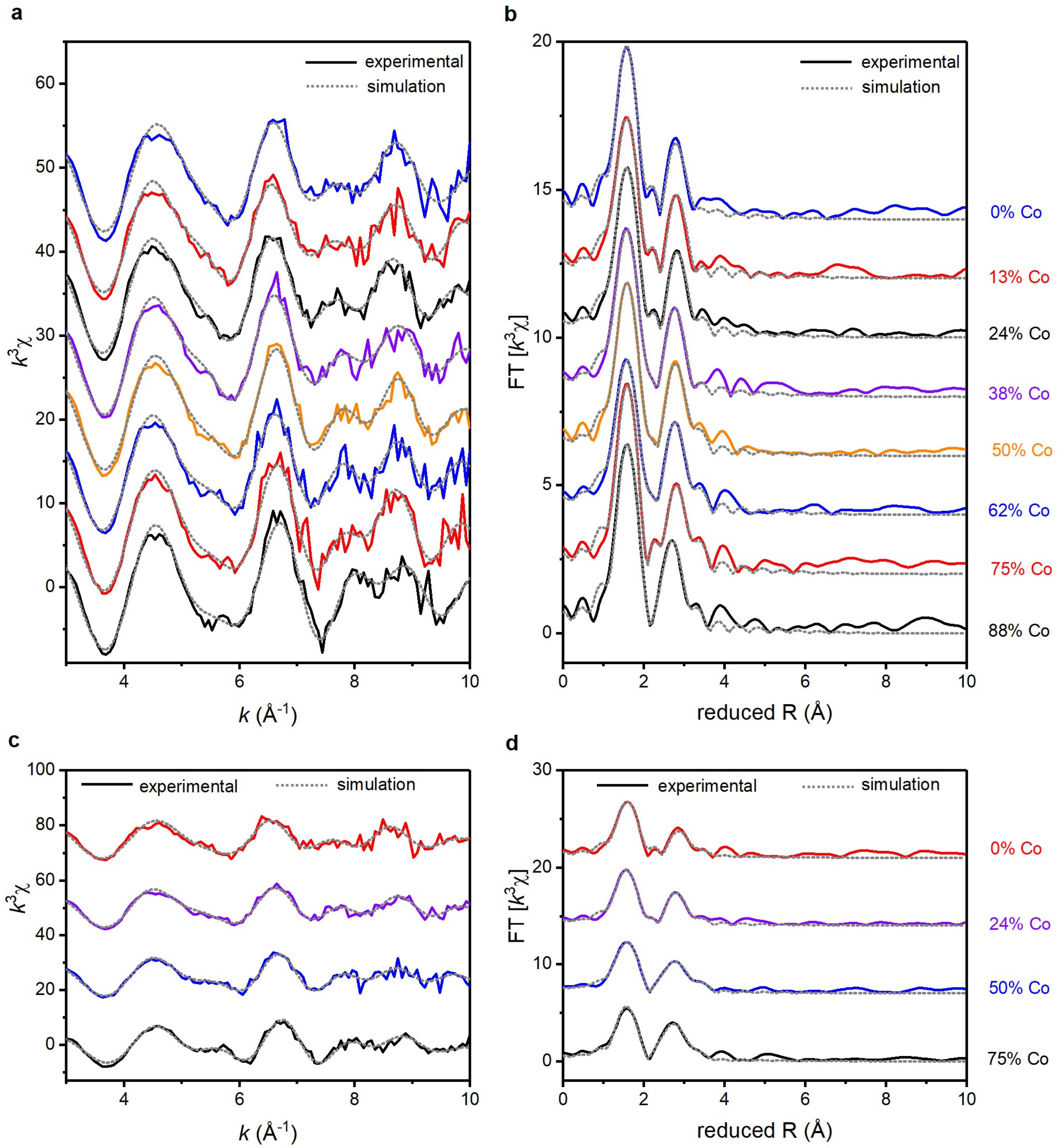

Supplementary Figure 5. X-ray absorptifon fine-structure spectra on the Fe K-edge. a, Experimental data and simulation results in *k*-space and their Fourier transforms **a,b,** for as-prepared films and **c,d,** for *quasi in-situ* films frozen at 1.5 V vs. RHE.

Supplementary Table 3. EXAFS fit parameters of the Co K-edge for the composition series[a]

Composition	Co-O		Co-O		Co-Co		Co-Fe		Co-Co		
% Co	***R* (Å)**	***N***	***R* (Å)**	***N***	***R* (Å)**	***N***	***R* (Å)**	***N***	***R* (Å)**	***N***	**R_f (%)**
100[b]	1.905 (0.004)	4.11 (0.16)	2.068 (0.014)	1.25 (0.18)	2.831 (0.003)	2.09 (0.10)	-	-	3.40 (0.008)	1.18 (0.16)	10.9
88[b]	1.904 (0.003)	4.20 (0.15)	2.077 (0.012)	1.37 (0.18)	2.826 (0.005)	2.58 (0.20)	2.988 (0.017)	0.99 (0.23)	3.412 (0.012)	0.84 (0.20)	7.0
75[b]	1.914 (0.003)	3.97 (0.11)	2.096 (0.007)	1.80 (0.14)	2.842 (0.004)	2.38 (0.15)	3.025 (0.008)	1.43 (0.20)	3.385 (0.017)	0.52 (0.16)	8.8
62[b]	1.909 (0.003)	4.02 (0.15)	2.081 (0.011)	1.59 (0.18)	2.840 (0.006)	2.17 (0.18)	3.008 (0.012)	1.23 (0.23)	3.44 (0.012)	0.82 (0.19)	10.4
50[b]	1.911 (0.005)	3.68 (0.19)	2.079 (0.013)	1.74 (0.22)	2.855 (0.008)	2.00 (0.25)	3.025 (0.015)	1.31 (0.32)	3.401 (0.036)	0.383 (0.25)	15.5
38[c]	1.906 (0.006)	3.85 (0.22)	2.081 (0.016)	1.73 (0.25)	2.843 (0.011)	2.09 (0.29)	3.018 (0.018)	1.64 (0.33)	3.489 (0.033)	0.476 (0.28)	11.2
24[c]	1.923 (0.008)	2.61 (0.22)	2.084 (0.008)	3.04 (0.24)	2.905 (0.012)	1.45 (0.25)	3.079 (0.011)	1.89 (0.29)	3.66 (0.043)	0.33 (0.24)	19.7
13[c]	1.921 (0.006)	3.30 (0.21)	2.101 (0.011)	2.37 (0.25)	2.932 (0.015)	1.48 (0.31)	3.109 (0.019)	1.35 (0.36)	3.618 (0.045)	0.40 (0.27)	24.1

[a] E_0 = -1.50; S_0^2= 0.78; $2\sigma^2$ = 0.0055 (Co-O shells) or 0.0067 (Co-M shells)

[b] Fitting region: $3 \leq k$ ($Å^{-1}$) ≤ 12; R_f calculated between $1.0 \leq R$ (Å) ≤ 4.0

[c] Fitting region: $3 \leq k$ ($Å^{-1}$) ≤ 11; R_f calculated between $1.0 \leq R$ (Å) ≤ 4.0

Supplementary Table 4. EXAFS fit parameters of the Fe K-edge for the composition series[a]

Composition	Fe-O		Fe-O		Fe-Fe		Fe-Fe		Fe-Co		
% Co	***R* (A)**	***N***	***R* (A)**	***N***	***R* (A)**	*N*	*R* (A)	***N***	***R* (A)**	***N***	**R_f (%)**
88	2.005 (0.044)	4.5 (3.8)	1.911 (0.100)	1.9 (3.5)	3.031 (0.064)	3.1 (1.4)	3.348 (0.058)	0.9 (1.1)	2.879 (0.048)	2.5 (1.5)	7.8
75	1.972 (0.008)	5.6 (0.4)	2.183 (0.074)	0.7 (0.4)	3.068 (0.027)	4.6 (1.4)	3.391 (0.030)	1.8 (1.0)	2.882 (0.025)	3.0 (0.9)	6.8
62	1.932 (0.018)	3.8 (0.8)	2.075 (0.036)	2.3 (0.8)	3.049 (0.038)	3.9 (1.1)	3.391 (0.044)	1.2 (0.8)	2.879 (0.039)	2.2 (1.1)	5.5
50	1.935 (0.019)	4.0 (1.2)	2.053 (0.037)	2.5 (1.1)	3.077 (0.031)	3.4 (0.9)	3.409 (0.026)	1.4 (0.6)	2.915 (0.026)	2.4 (0.8)	7.4
38	1.936 (0.020)	4.2 (1.0)	2.070 (0.042)	2.4 (1.0)	3.059 (0.036)	3.9 (1.4)	3.358 (0.130)	0.3 (1.0)	2.892 (0.038)	2.2 (1.1)	11.2
24	1.942 (0.023)	4.4 (1.5)	2.076 (0.063)	2.0 (1.2)	3.068 (0.043)	3.9 (1.6)	3.415 (0.099)	0.8 (1.1)	2.885 (0.052)	1.9 (1.2)	6.4
13	1.925 (0.020)	3.8 (1.0)	2.057 (0.032)	2.8 (0.9)	3.036 (0.013)	3.7 (0.6)	3.632 (0.13)	0.3 (0.4)	2.835 (0.028)	1.3 (0.5)	8.5
0	1.963 (0.011)	4.9 (0.5)	2.131 (0.084)	0.8 (0.5)	3.021 (0.012)	1.9 (0.4)	3.501 (0.039)	0.9 (0.5)	-	-	10.5

[a] E_0 = +3.25; S_0^2= 0.78; $2\sigma^2$ = 0.011 (Fe-O shells) or 0.012 (Fe-M shells)

Fitting region: $3 \leq k$ (Å^{-1}) ≤ 10; R_f calculated between $1.0 \leq R$ (Å) ≤ 4.0

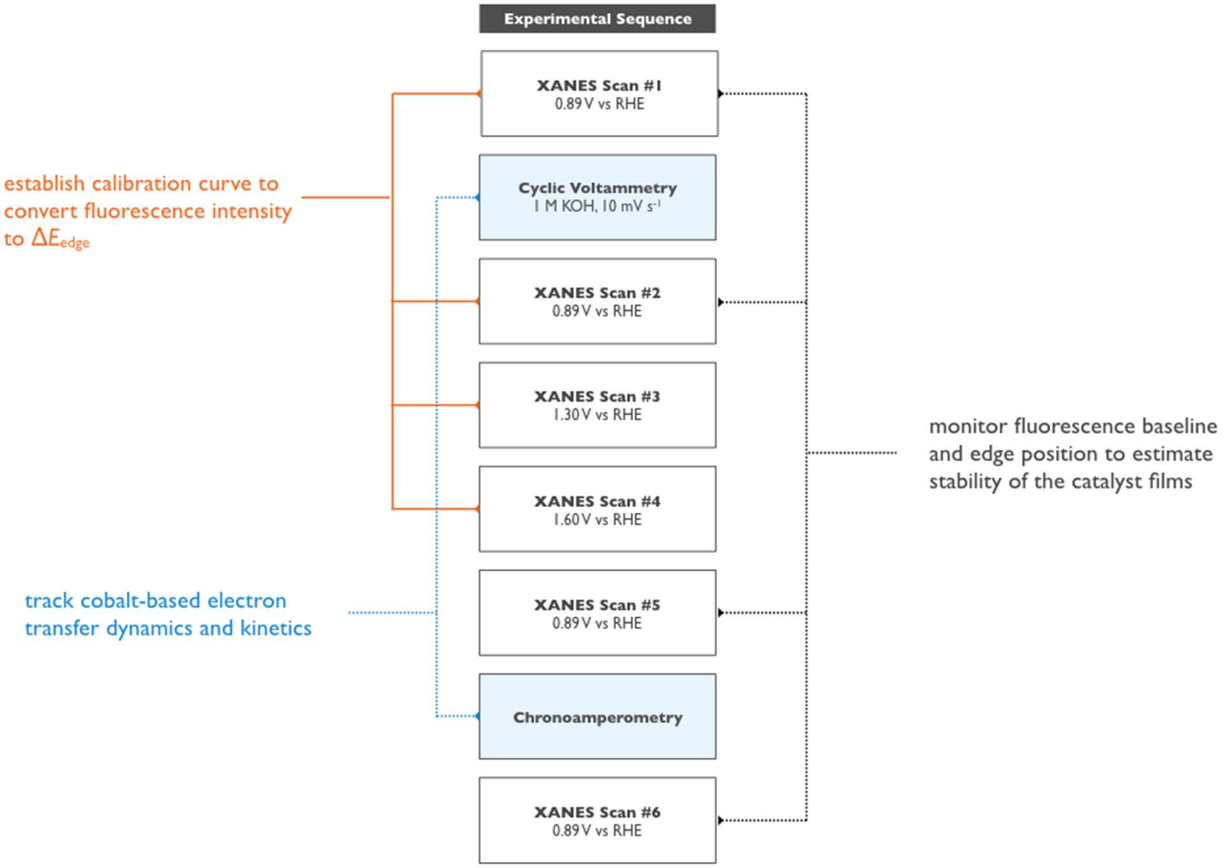

Supplementary Figure 6. Experimental design for *in-situ* X-ray absorption spectroscopy experiments. Individual electrodes **(100% Co, 88% Co, 75% Co, 62% Co** and **50% Co)** were sequentially examined with no pauses between experimental steps.

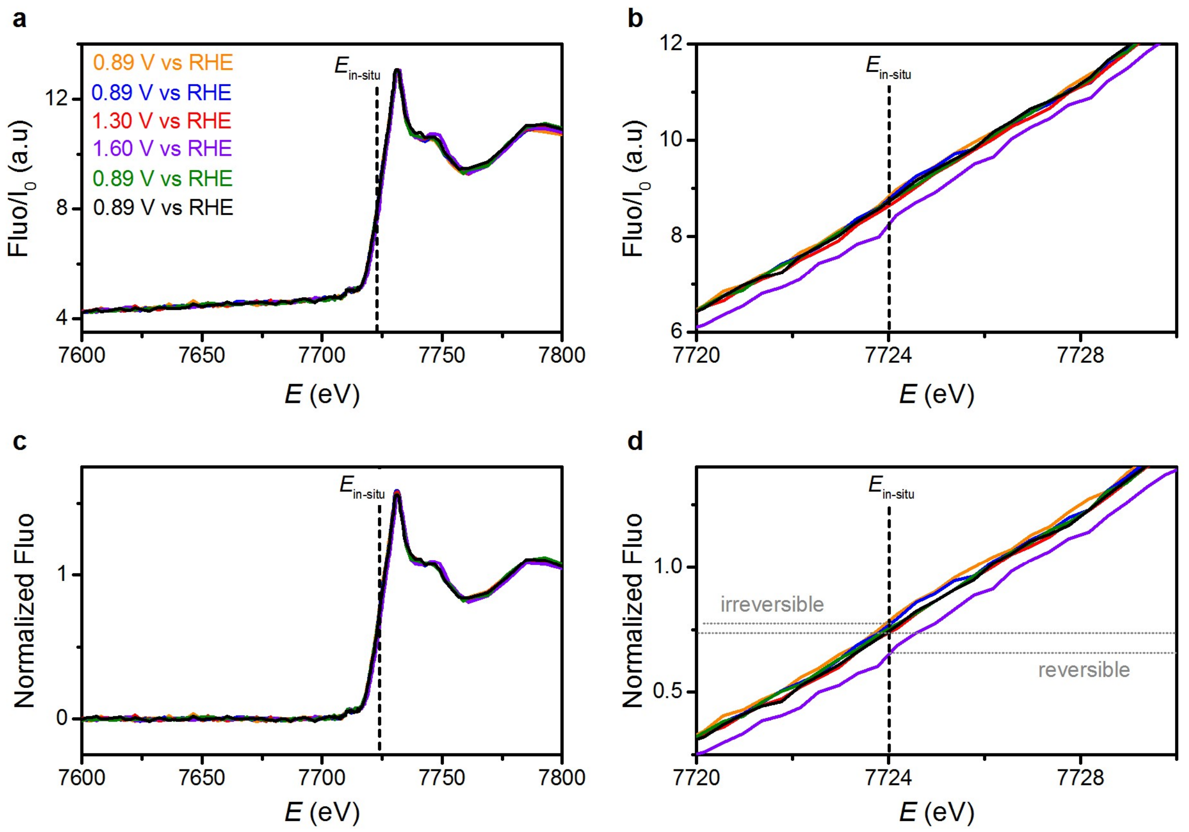

Supplementary Figure 7. *In-situ* Co K-edge X-ray absorption near-edge spectra acquired on 100% Co. a, b, Raw fluorescence data acquired during the six chronoamperometric experiments. **c, d,** The same spectra following baseline correction and normalization. The incident energy utilized during CV experiments is indicated by $E_{\text{in-situ}}$.

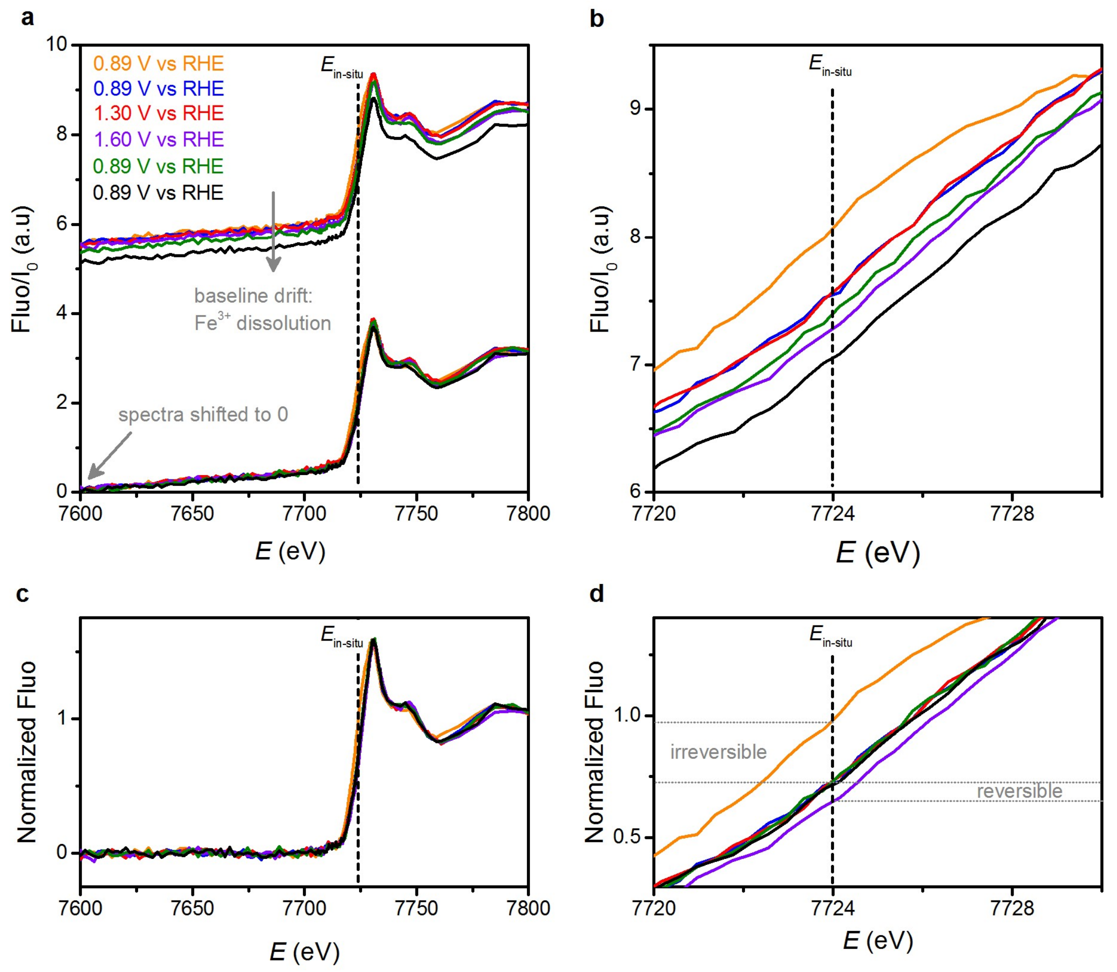

Supplementary Figure 8. ***In-situ*** **Co K-edge X-ray absorption near-edge spectra acquired on 50% Co. a, b,** Raw fluorescence data acquired during the six chronoamperometric experiments. **c, d,** The same results following baseline correction and normalization. The role of Fe-dissolution is readily observed in the lower data set in panel **a**, where the spectra were simply shifted to begin at Fluo/I_0 = 0. Comparison of panels **b** and **d** reveal experimental complications that arise due to Fe loss. The incident energy utilized during CV experiments is indicated by $E_{\text{in-situ}}$.

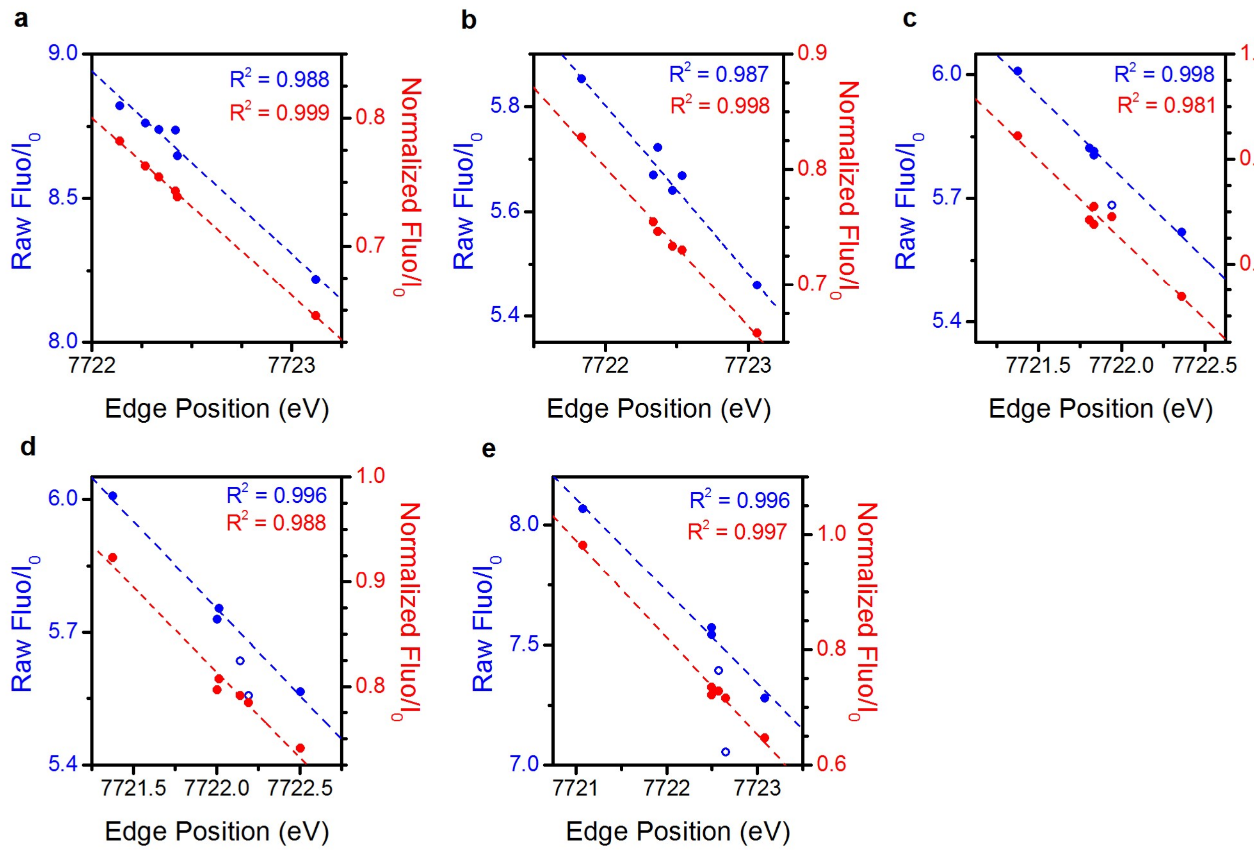

Supplementary Figure 9. Calibration curves for conversion of fluorescence intensity to Co K-edge location for *in-situ* XAS experiments. Calibration curves and linear regression fits for raw and processed spectra: **a,** 100% Co, **b,** 88% Co, **c,** 75% Co, **d,** 62% Co, **e,** 50% Co. Hollow data points represent the final two XANES spectra where significant Fe dissolution introduced inaccuracies and were not used in the calibration.

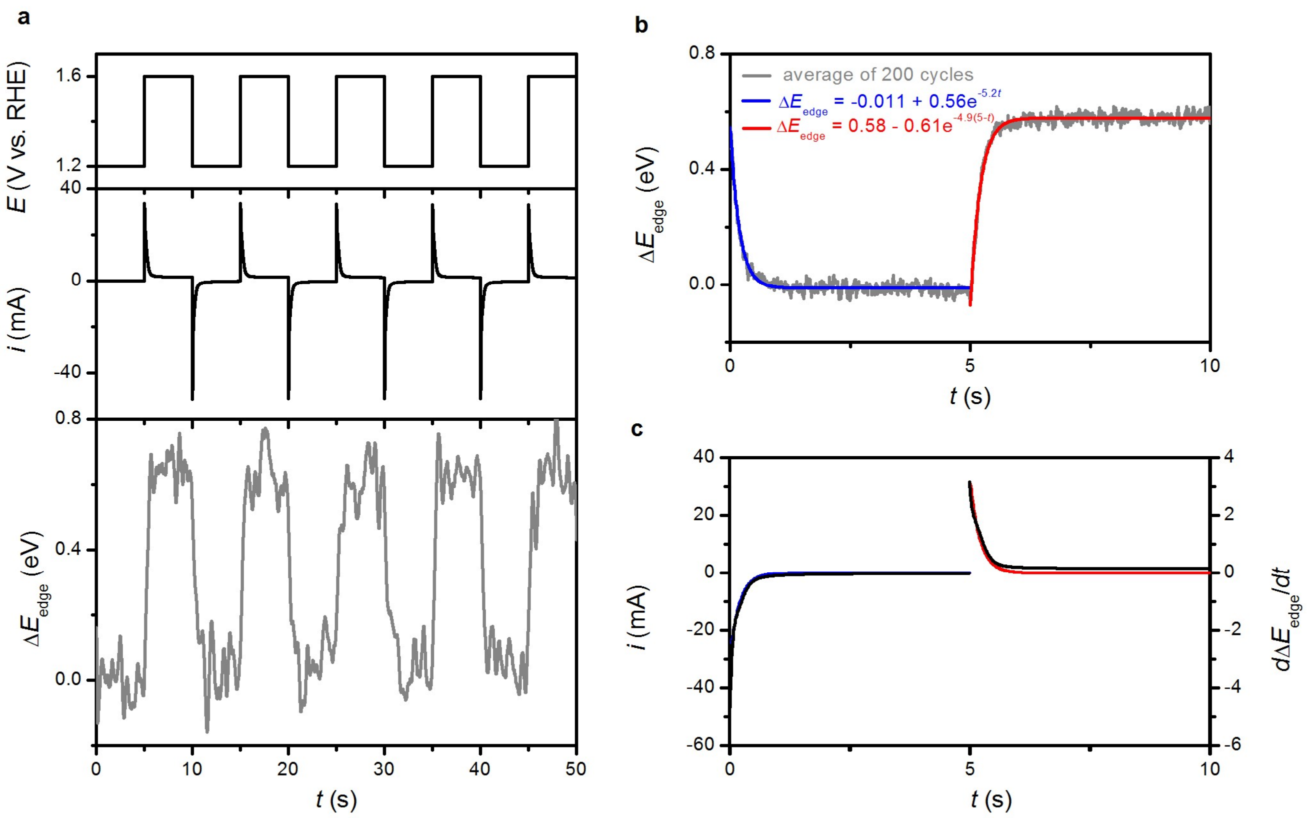

Supplementary Figure 10. ***In-situ* XAS-chronoamperometry on 100% Co. a.** The voltage step protocol and resultant electrochemical current and spectroscopic response for the initial 5 voltage step cycles. A total of 200 cycles were performed. **b.** Spectroscopic response obtained by averaging all voltage cycles; exponential fit lines included for the anodic and cathodic components. **c.** The current response (average of all cycles) compared to the derivative of the exponential fit for the anodic and cathodic components.

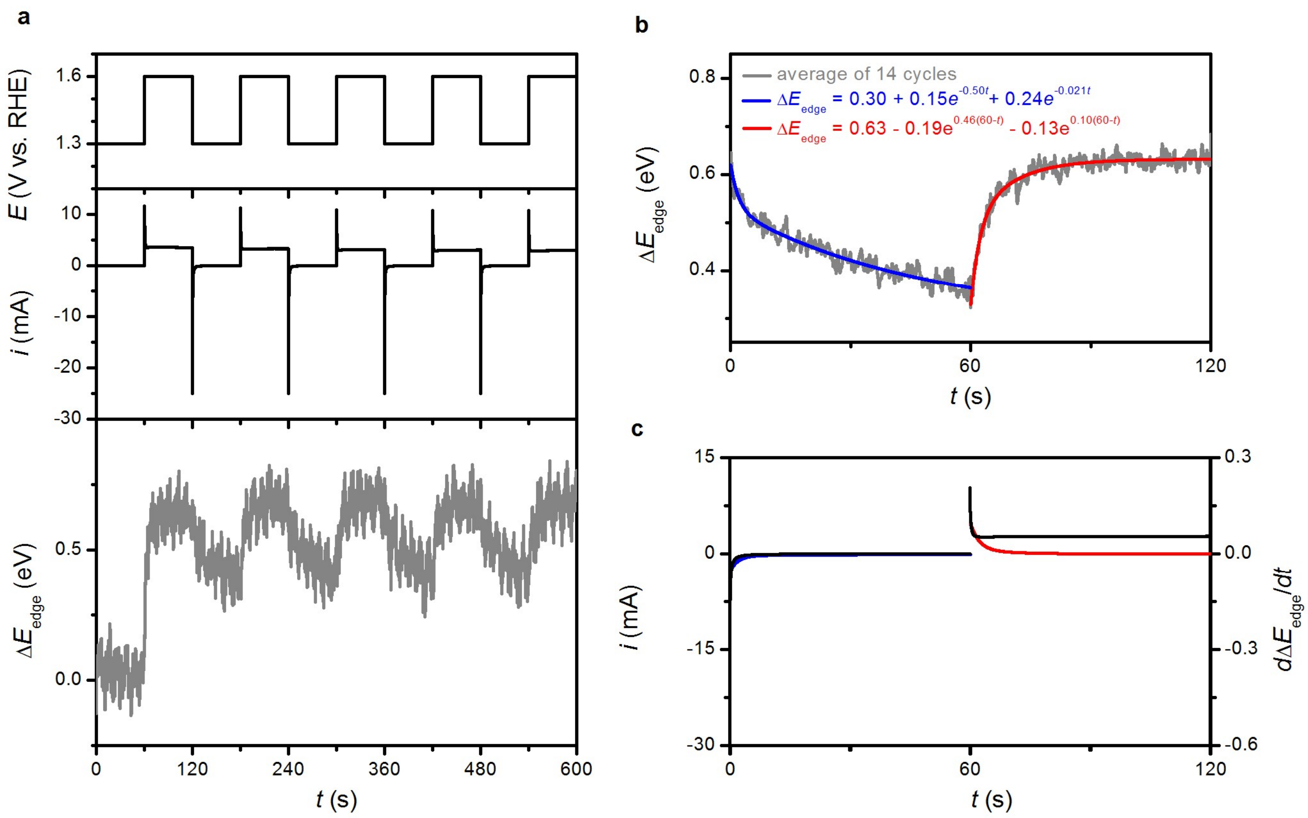

Supplementary Figure 11. ***In-situ* XAS-chronoamperometry on 88% Co.** The voltage step protocol and resultant electrochemical current and spectroscopic response for the initial 5 voltage step cycles. A total of 15 cycles were performed. **b.** Spectroscopic response obtained by averaging all voltage cycles; biexponential fit lines included for the anodic and cathodic components. **c.** The current response (average of all cycles) compared to the derivative of the exponential fit for the anodic and cathodic components.

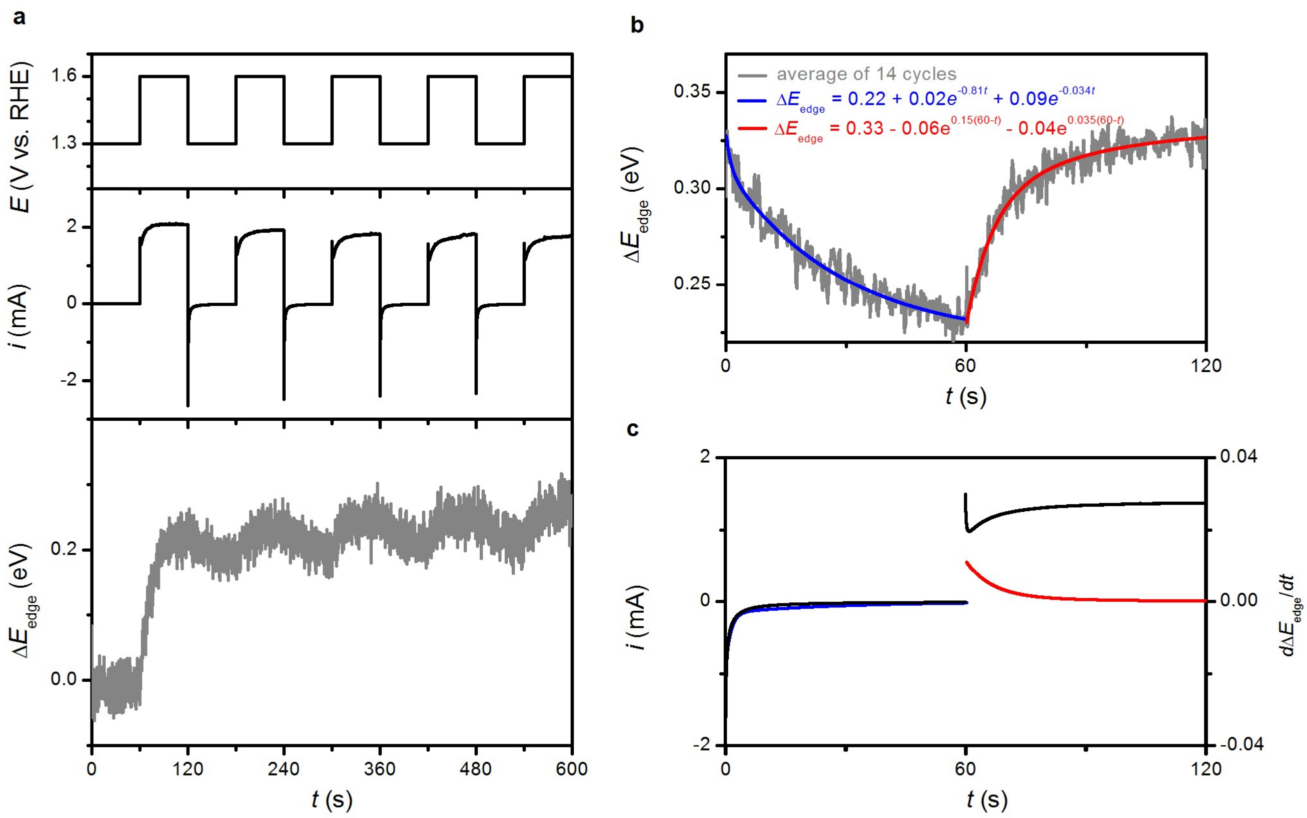

Supplementary Figure 12. *In-situ* XAS-chronoamperometry on 75% Co. The voltage step protocol and resultant electrochemical current and spectroscopic response for the initial 5 voltage step cycles. A total of 15 cycles were performed. **b.** Spectroscopic response obtained by averaging all voltage cycles; biexponential fit lines included for the anodic and cathodic components. **c.** The current response (average of all cycles) compared to the derivative of the exponential fit for the anodic and cathodic components.

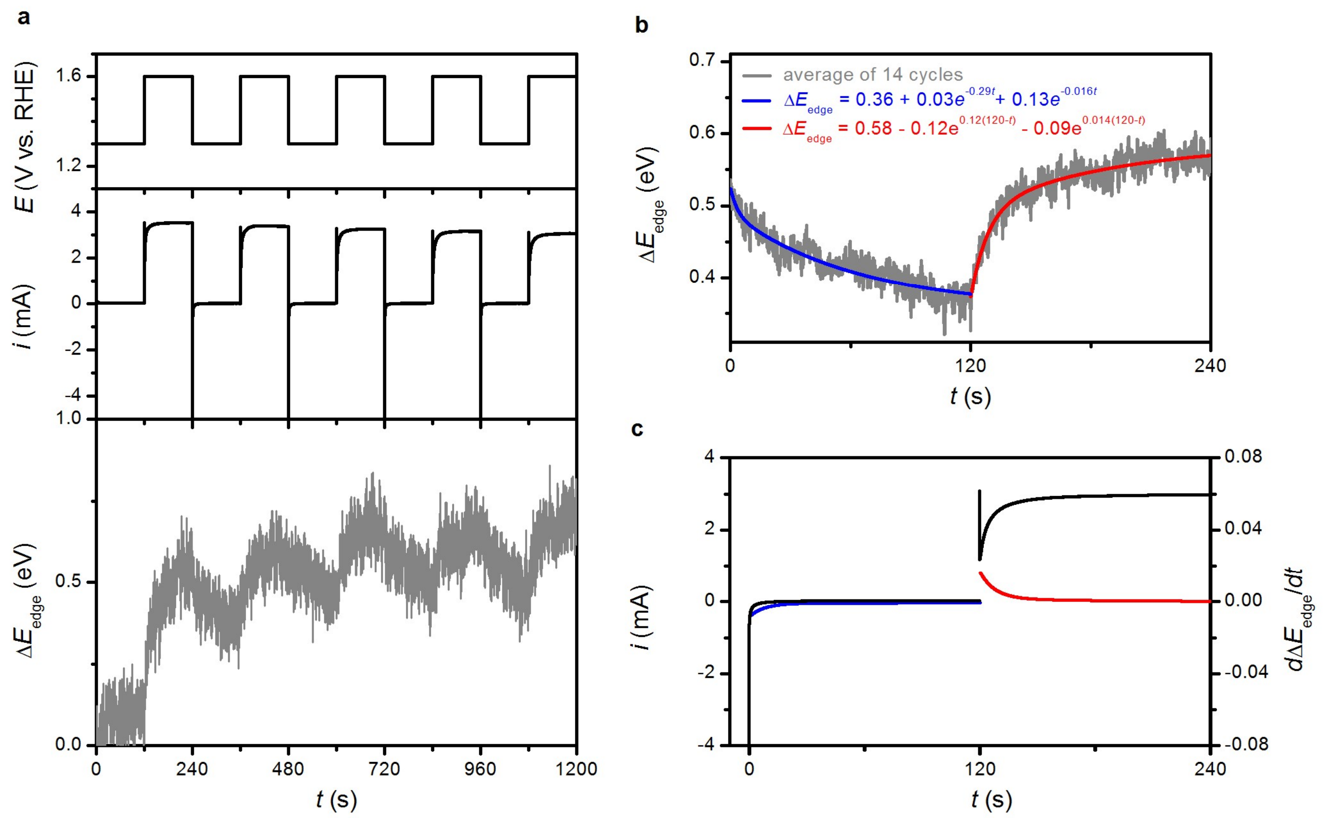

Supplementary Figure 13. *In-situ* XAS-chronoamperometry on 62% Co. The voltage step protocol and resultant electrochemical current and spectroscopic response for the initial 5 voltage step cycles. A total of 15 cycles were performed. **b.** Spectroscopic response obtained by averaging all voltage cycles; biexponential fit lines included for the anodic and cathodic components. **c.** The current response (average of all cycles) compared to the derivative of the exponential fit for the anodic and cathodic components.

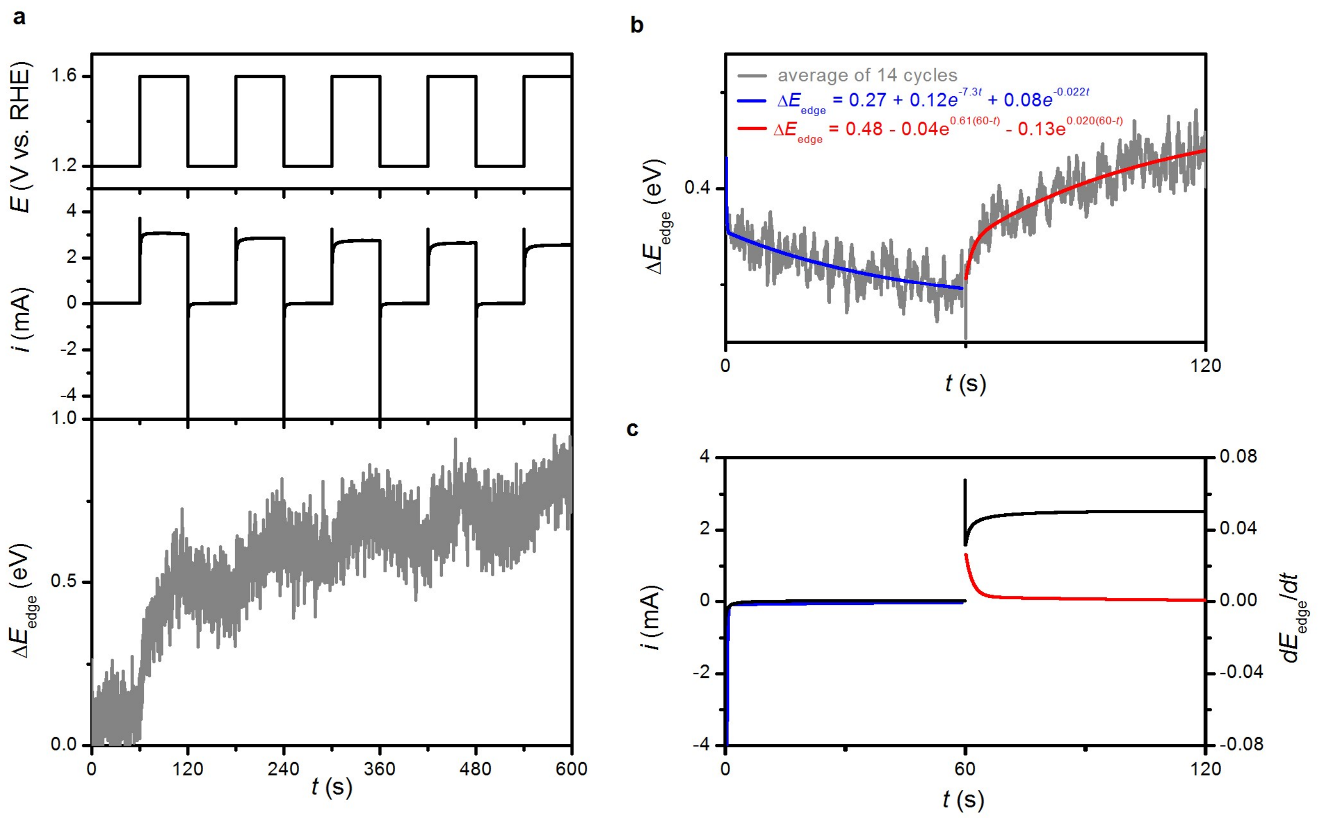

Supplementary Figure 14. *In-situ* XAS-chronoamperometry on 50% Co. The voltage step protocol and resultant electrochemical current and spectroscopic response for the initial 5 voltage step cycles. A total of 15 cycles were performed. **b.** Spectroscopic response obtained by averaging all voltage cycles; bi-exponential fit lines included for the anodic and cathodic components. **c.** The current response (average of all cycles) compared to the derivative of the exponential fit for the anodic and cathodic components.

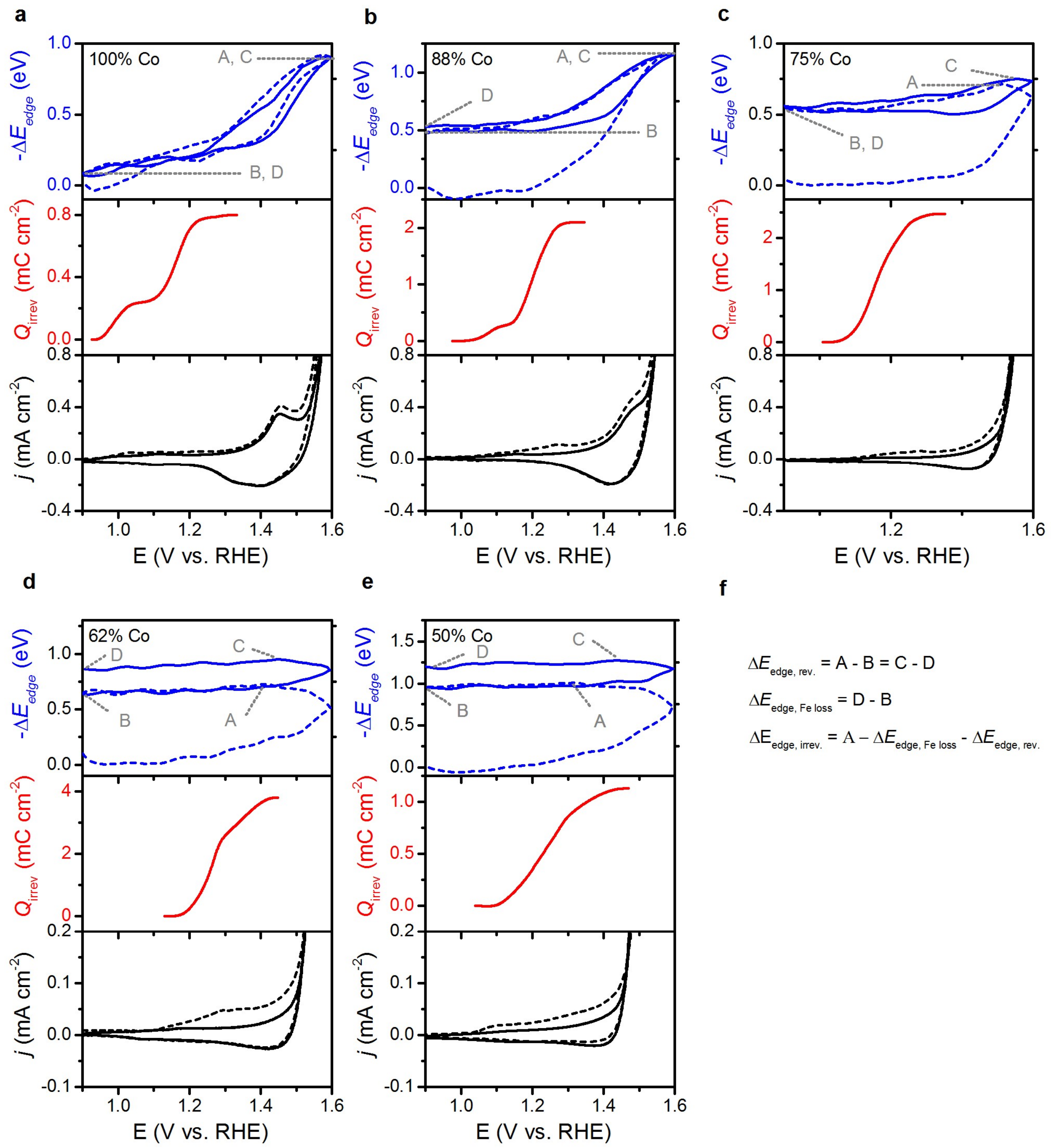

Supplementary Figure 15. The shift in Co K-edge position, the irreversible charge passed, and the electrochemical current density recorded during *in-situ* XAS-CV experiments. a-e, Changes in the Co K-edge position, integral of the irreversible charge passed, and current density for the initial two CV cycles. **f,** Calculations for deconvolution of reversible and irreversible contributions to oxidation state change. Irreversible charge passed was calculated by integration of the difference CVs (see main text).

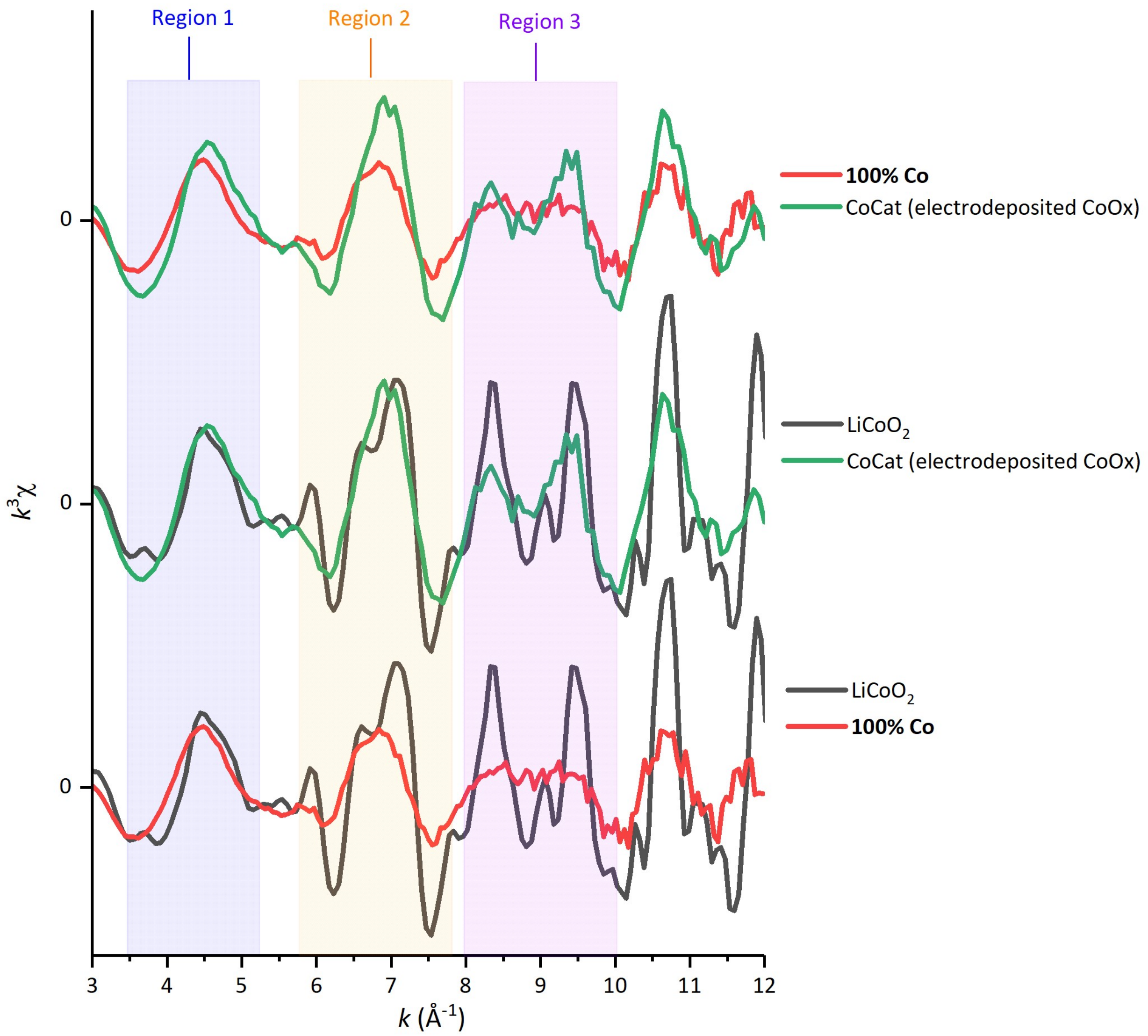

Supplementary Figure 16. Comparisons between the X-ray absorption fine-structure spectra. A comparison of the EXAFS oscillations for **100% Co** with $LiCoO_2$ and CoCat. Regions are labeled to highlight distinct structural differences.

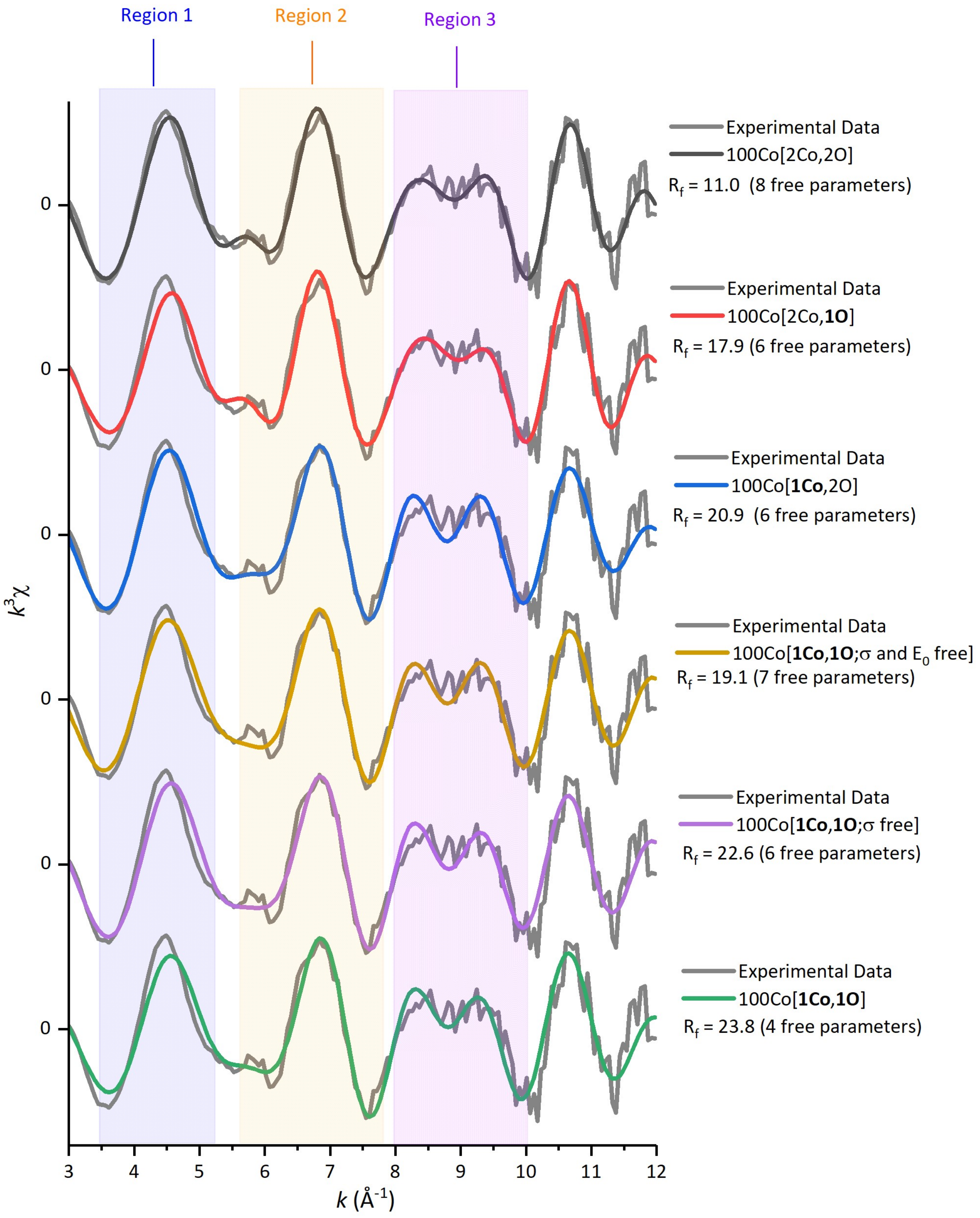

Supplementary Figure 17. Alternative simulations considered for 100% Co. Fit parameters for a representative selection of alternative simulation models considered for **100% Co.** Changes relative to the model in Supplementary Table 3 are bolded and labels are defined in Supplementary Table 5.

Supplementary Table 5: Fit parameters for selected alternative models for the 100% Co sample[a]

Label[b]		Co-O	Co-O	Co-Co	Co-Co	R-factor
100Co [2Co,2O]	*R*	1.905 (0.004)	2.068 (0.014)	2.831 (0.003)	3.400 (0.008)	10.9
	N	4.11 (0.16)	1.25 (0.18)	2.09 (0.10)	1.18 (0.16)	
	σ	*0.0524**	*0.0524**	*0.0579**	*0.0579**	
100Co [2Co,**1O**]	*R*	1.916 (0.003)		2.831 (0.004)	3.404 (0.011)	17.9
	N	3.76 (0.16)		2.03 (0.13)	1.12 (0.22)	
	σ	*0.0524**		*0.0579**	*0.0579**	
100Co [**1Co**,2O]	*R*	1.904 (0.014)	2.056 (0.057)	2.829 (0.009)		20.9
	N	3.97 (0.59)	1.23 (0.67)	2.08 (0.33)		
	σ	*0.0524**	*0.0524**	*0.0579**		
100Co **[1Co,1O]** σ and E_0 free	*R*	1.904 (0.01)		2.816 (0.009)	*E_0 = -4.36 eV*	19.1
	N	4.84 (0.64)		1.73 (0.58)	*S_0^2 = 0.787*	
	σ	0.072 (0.009)		0.048 (0.023)		
100Co **[1Co,1O]** σ free	*R*	1.918 (0.005)		2.827 (0.005)		22.6
	N	4.67 (0.50)		1.78 (0.48)		
	σ	0.071 (0.008)		0.050 (0.016)		
100Co **[1Co,1O]**	*R*	1.917 (0.005)		2.829 (0.006)		23.8
	N	3.75 (0.24)		2.03 (0.20)		
	σ	*0.0524**		*0.0579**		

[a] E_0 = -1.50 and S_0^2= 0.78 unless otherwise mentioned; fitting region: $3 \leq k$ (Å^{-1}) ≤ 12; R_f calculated between $1.0 \leq R$ (Å) ≤ 4.0; values marked with a * were fixed during fitting

[b] Labels correspond to those in Supplementary Fig. 18; changes relative to the model in Supplementary Table 3 are bolded.

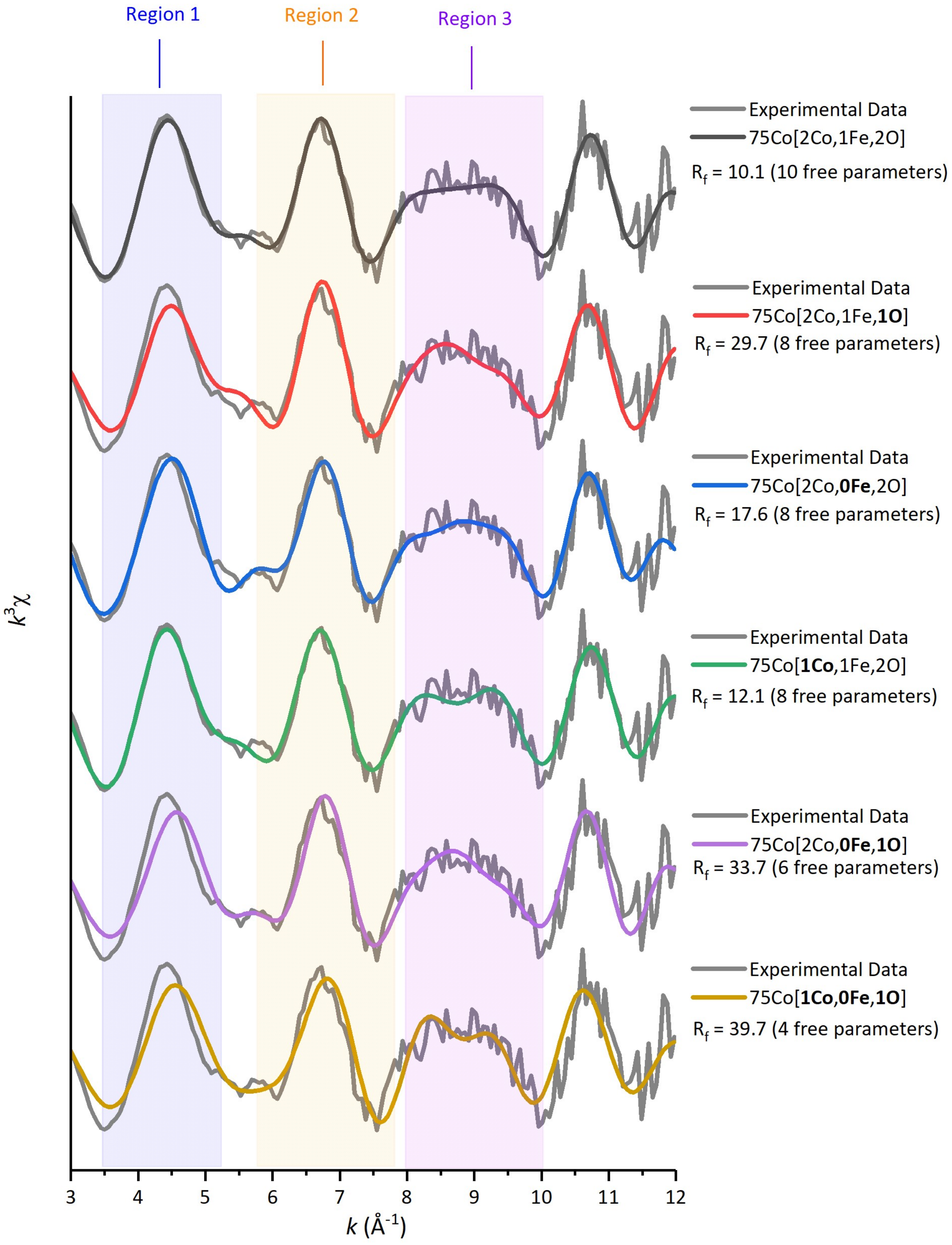

Supplementary Figure 18. Alternative simulations considered for 75% Co. Fit parameters for a representative selection of alternative simulation models considered for **75% Co.** Changes relative to the model in Supplementary Table 3 are bolded and labels are defined in Supplementary Table 6.

Supplementary Table 6: Fit parameters for selected alternative models for the 75% Co sample[a]

Labels[b]		Co-O	Co-O	Co-Co	Co-Fe	Co-Co	R-factor
75Co [2Co,1Fe,2O]	*R*	1.914 (0.003)	2.096 (0.007)	2.842 (0.004)	3.025 (0.008)	3.385 (0.017)	8.8
	N	3.97 (0.11)	1.80 (0.14)	2.38 (0.15)	1.43 (0.20)	0.52 (0.16)	
	σ	*0.0524**	*0.0524**	*0.0579**	*0.0579**	*0.0579**	
75Co [2Co,1Fe,**1O**]	*R*	1.927 (0.003)		2.831 (0.005)	3.001 (0.010)	3.411 (0.020)	29.7
	N	3.34 (0.13)		2.42 (0.22)	1.57 (0.28)	0.54 (0.22)	
	σ	*0.0524**		*0.0579**	*0.0579**	*0.0579**	
75Co [2Co,**0Fe**,2O]	*R*	1.917 (0.003)	2.104 (0.009)	2.840 (0.005)		3.383 (0.008)	17.6
	N	4.05 (0.16)	1.82 (0.20)	1.40 (0.11)		1.22 (0.17)	
	σ	*0.0524**	*0.0524**	*0.0579**		*0.0579**	
75Co [**1Co**,1Fe,2O]	*R*	1.914 (0.004)	2.091 (0.010)	2.843 (0.004)	3.023 (0.007)		12.1
	N	3.91 (0.16)	1.80 (0.20)	2.72 (0.19)	1.94 (0.23)		
	σ	*0.0524**	*0.0524**	*0.0579**	*0.0579**		
75Co [2Co,**0Fe**,**1O**]	*R*	1.924 (0.008)		2.836 (0.013)		3.381 (0.024)	33.7
	N	3.37 (0.36)		1.34 (0.30)		1.12 (0.47)	
	σ	*0.0524**		*0.0579**		*0.0579**	
75Co [**1Co**,**0Fe**,**1O**]	*R*	1.926 (0.008)		2.833 (0.013)			39.7
	N	3.37 (0.36)		1.31 (0.30)			
	σ	*0.0524**		*0.0579**			

[a] E_0 = -1.50 and S_0^2= 0.78 unless otherwise mentioned; fitting region: $3 \leq k$ (Å^{-1}) ≤ 12; R_f calculated between $1.0 \leq R$ (Å) ≤ 4.0; values marked with a * were fixed during fitting

[b] Labels correspond to those in Supplementary Fig. 18; changes relative to the model in Supplementary Table 3 are bolded.

Supplementary References

1. Risch, M. *et al.* Water oxidation by electrodeposited cobalt oxides-role of anions and redox-inert cations in structure and function of the amorphous catalyst. *ChemSusChem* **5,** 542–549 (2012).
2. Risch, M. *et al.* Water oxidation by amorphous cobalt-based oxides: *in situ* tracking of redox transitions and mode of catalysis. *Energy Environ. Sci.* **8,** 661–674 (2015).
3. Dau, H., Liebisch, P. & Haumann, M. X-ray absorption spectroscopy to analyze nuclear geometry and electronic structure of biological metal centers - potential and questions examined with special focus on the tetra-nuclear manganese complex of oxygenic photosynthesis. *Anal. Bioanal. Chem.* **376,** 562–583 (2003).
4. Risch, M. *et al.* Cobalt−oxo core of a water-oxidizing catalyst film. *J. Am. Chem. Soc.* **131,** 6936–6937 (2009).
5. Kanan, M. W. *et al.* Structure and valency of a cobalt−phosphate water oxidation catalyst determined by in situ X-ray spectroscopy. *J. Am. Chem. Soc.* **132,** 13692–13701 (2010).
6. González-Flores, D. *et al.* Heterogeneous water oxidation: Surface activity versus amorphization activation in cobalt phosphate catalysts. *Angew. Chem. Int. Ed.* **54,** 2472–2476 (2015).
7. Trudel, S., Daryl Crozier, E., Gordon, R. A., Budnik, P. S. & Hill, R. H. X-ray absorption fine structure study of amorphous metal oxide thin films prepared by photochemical metalorganic deposition. *J. Solid State Chem.* **184,** 1025–1035 (2011).
8. Friebel, D. *et al.* Identification of highly active Fe sites in (Ni,Fe)OOH for electrocatalytic water splitting. *J. Am. Chem. Soc.* **137,** 1305–1313 (2015).
9. Kurzman, J. A., Dettelbach, K. E., Martinolich, A. J., Berlinguette, C. P. & Neilson, J. R. Structural characteristics and eutaxy in the photo-deposited amorphous iron oxide oxygen evolution catalyst. *Chem. Mater.* **27,** 3462–3470 (2015).
